# Chronic stress unleashes an intratumor phage-fibroblast-B cell circuit to promote tumor growth

**DOI:** 10.1016/j.ccell.2026.06.004

**Published:** 2026-06-25

**Authors:** Hilal Bashir, Katherine Z. Sanidad, Purnima Ravisankar, Kelly M. Banks, Avipsa Bose, Shui Yu, Joshua J.C. McGrath, Hannah C. Carrow, Taeyul K. Kim, Chloe A. Troxell, Julia A. Brown, Lucy R. Hart, Jeremy Goc, Mikala Egeblad, Jayanta Chaudhuri, Han Sang Kim, Patrick C. Wilson, David C. Lyden, Irina R. Matei, Naohiro Inohara, Gregory F. Sonnenberg, Melody Y. Zeng

**Affiliations:** 1 Gale and Ira Drukier Institute for Children’s Health, Weill Cornell Medicine, New York, NY 10065, USA; 2 Department of Pediatrics, Weill Cornell Medicine, New York, NY 10065, USA; 3 Immunology and Microbial Pathogenesis Program, Weill Cornell Graduate School, New York, NY 10065, USA; 4 Department of Internal Medicine, Graduate School of Medical Science, Brain Korea 21 FOUR Project, Yonsei University College of Medicine, Seoul, Republic of Korea; 5 Jill Roberts Institute for Research in Inflammatory Bowel Disease, Weill Cornell Medicine, New York, NY 10065, USA; 6 Department of Medicine, Division of Gastroenterology & Hepatology, Weill Cornell Medicine, New York, NY 10065, USA; 7 Department of Cell Biology, Johns Hopkins School of Medicine, Baltimore, MD 21202, USA; 8 Immunology Program of the Sloan Kettering Institute, Memorial Sloan Kettering Cancer Center, New York, NY 10065, USA; 9 Yonsei Cancer Center, Division of Medical Oncology, Department of Internal Medicine, Yonsei University College of Medicine, Seoul, Republic of Korea; 10 Meyer Cancer Center, Weill Cornell Medicine, New York, NY 10065, USA; 11 Rogel Cancer Center, University of Michigan, Ann Arbor, MI 48109, USA; 12 Lead contact

## Abstract

Chronic stress disrupts the gut microbiota in patients with cancer; however, how stress-induced microbiota perturbations impact anti-tumor immunity remains unclear. Here, we show that the gut microbiota is required for chronic stress-induced glucocorticoid production, which impairs antigen-specific germinal center B cell responses. In mouse models of colorectal cancer or melanoma, chronic stress promotes translocation of a gut pathobiont, *Enterococcus gallinarum* (Eg), to tumors. Within tumors, Eg phage DNA induces glucocorticoid production by cancer-associated fibroblasts (CAFs) via TLR9, which suppresses anti-tumor B cell responses through the glucocorticoid receptor. Targeting intratumoral TLR9 or Eg significantly lowers intratumor glucocorticoid levels and reverses the tumor-promoting effects of chronic stress. Extending these findings to human cancer, we identify lytic phages in a *Klebsiella pneumoniae* isolate from human colorectal tumors that promote tumor growth and detect phage DNA in human brain tumors. Together, our study reveals a chronic stress-induced intratumor phage-CAF-B cell circuit that weakens anti-tumor immunity.

## Graphical Abstarct

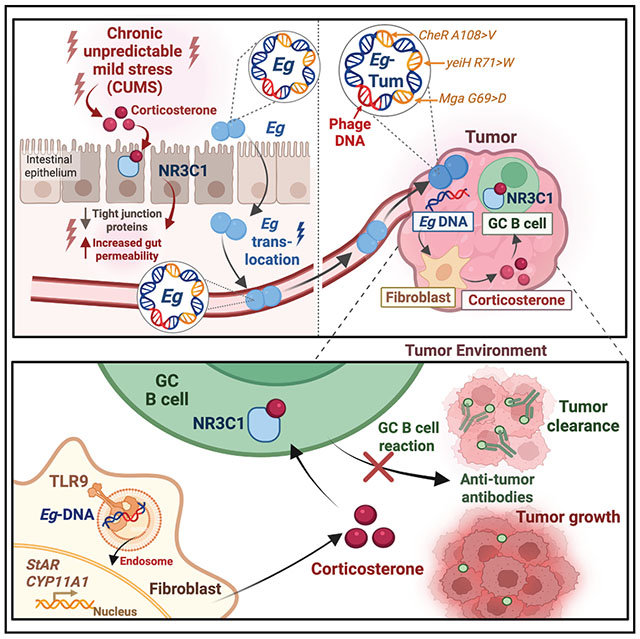

## In brief

Bashir et al. show that chronic stress disrupts the gut barrier, enabling *Enterococcus gallinarum* translocation to tumors. There, Eg phage DNA activates cancer-associated fibroblasts via TLR9 to produce local glucocorticoids that suppress germinal-center B cell responses and accelerate tumor growth. Targeting this phage-CAF-B cell circuit restores anti-tumor immunity.

## INTRODUCTION

Chronic stress is associated with increased metastasis and poor prognosis in patients with cancer.^1–4^ Through the hypothalamic-pituitary-adrenal (HPA) axis and the sympathetic nervous system, chronic stress induces the production of stress hormones, glucocorticoids, by the adrenal cortex. However, glucocorticoids can be synthesized by cells outside of the HPA axis, including thymic or intestinal epithelial cells and tumor cells,^5,6^ although regulatory mechanisms remain elusive. The glucocorticoid receptor (GR; encoded by *Nr3c1*) is a nuclear receptor expressed in many cell types, including immune cells and neurons. Glucocorticoids have pleiotropic effects on different T cell populations during development and activation,^7^ while elevated glucocorticoid levels disrupt the gut microbiota and promote inflammatory monocytes through direct signaling to enteric glia.^8,9^ The regulation and function of glucocorticoid signaling within tumors, however, remains poorly understood.

The gut microbiota has emerged as a tractable target that can be coupled with immunotherapies to improve cancer treatment outcomes.^10–13^ It remains unknown, however, how chronic stress-induced perturbation of the gut microbiota affects the anti-tumor immune response. Recent studies underscore a critical role for B cells in anti-tumor immunotherapy.^14–17^ Anti-tumor antibody responses require a robust intratumor germinal-center B cell response that is sustained by intratumor tertiary lymphoid structures.^14,18,19^ The B cell repertoire is heavily shaped by microbiota exposure^20,21^; however, the role of the gut microbiota in the regulation of intratumor B cell response remains undefined. Here, we employed mouse models of chronic stress coupled with colorectal or melanoma cancer models, as well as analysis of human colorectal tumors, to explore the interactions among chronic stress, the gut microbiota, and anti-tumor immunity. Our study has identified a gut microbiota-phage-B cell transkingdom circuit that could be a therapeutic target to reduce intratumor glucocorticoids and boost anti-tumor immunity.

## RESULTS

### Chronic stress accelerates tumor progression by dampening germinal center B cell response

**Figure 1.**
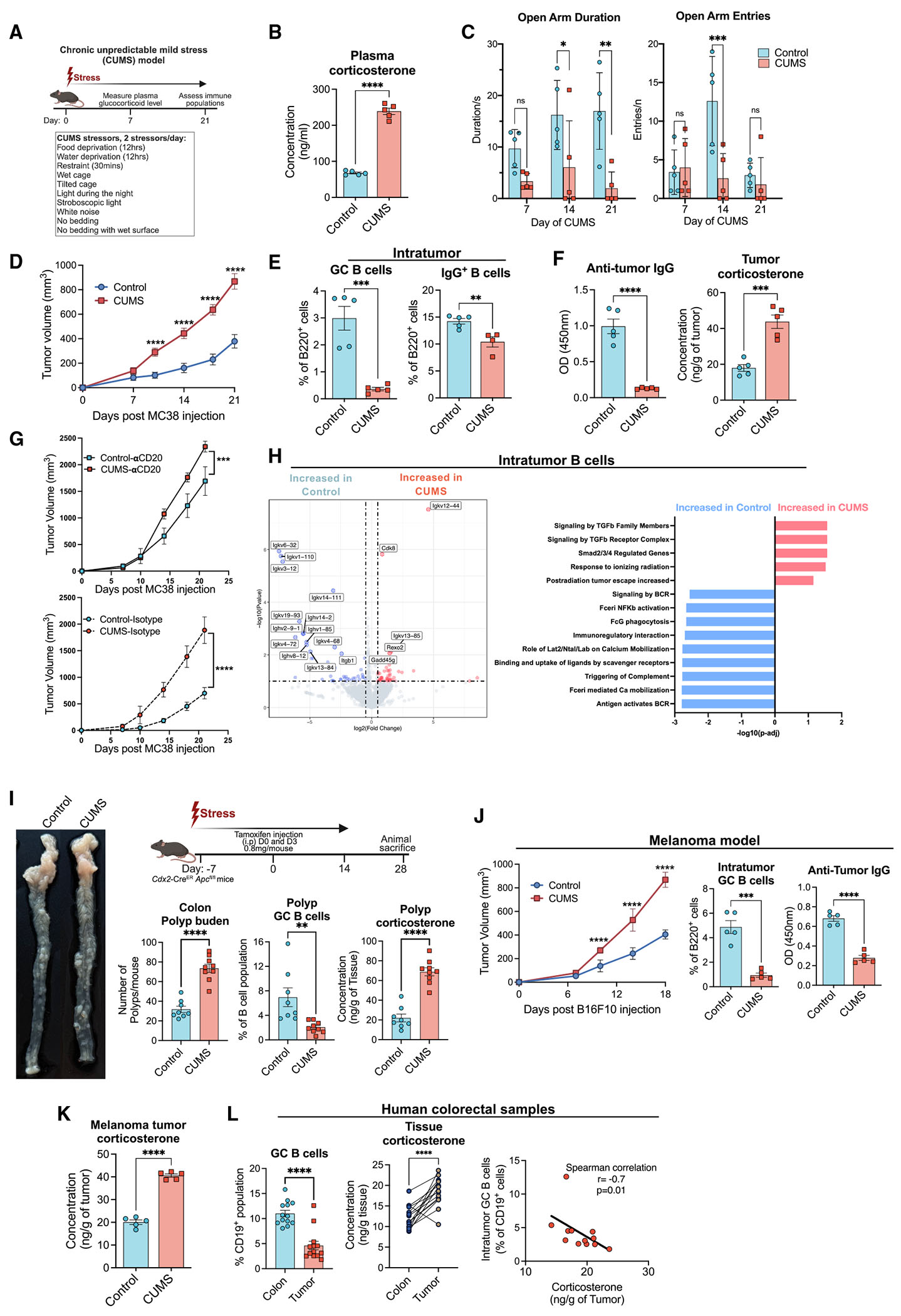
Chronic stress accelerates tumor progression by dampening anti-tumor B cell response (A) Chronic unpredictable mild stress model (CUMS). (B) Plasma corticosterone on CUMS day 7 (*n* = 5 mice/group). (C) Elevated plus maze (EPM) assessment at CUMS day 7, 14, or 21 (*n* = 5 mice/group). (D) MC38 tumor growth (n = 5mice/group). (E) Intratumor GC and IgG^+^ B cells 21 days post MC38 cell injection (*n* = 5 mice/group). (F) Plasma anti-tumor IgG (left) and tumor corticosterone (right) 21 days post-MC38 cell injection (*n* = 5 mice/group). (G) MC38 tumor growth in control and CUMS mice treated with anti-CD20 or isotype (*n* = 5 mice/group). At day 21, 5 mice from the CUMS+ anti-CD20 group and 1 mouse from the CUMS group without signs of distress showed tumor volume exceeding 2000 mm^3^ and were euthanized immediately. (H) Differential gene abundance (left) and differential signaling pathways (right) in control vs. CUMS intratumor B cells (*n* = 2 mice/group). (I) Polyp burdens in colons, and polyp GC B cells and corticesterone levels in control or CUMS *Cdx2*-Cre^ER^;*Apc*^fl/fL^ mice 28 days after tamoxifen injection (n = 8–9 mice/group). (J and K) Control or CUMS mice were inoculated subcutaneously with B16F10 melanoma. (J) Tumor growth (left, *n* = 5), intratumor GC B cells (middle), plasma anti-tumor IgG (right), and (K) intratumor corticosterone 21 days post tumor inoculation (*n* = 5 mice/group). (L) GC B cells (left), tissue corticosterone (middle), and Spearman’s correlation of tissue corticosterone with GC B cells (right) in human colon tumors and adjacent healthy tissue (*n* = 13 patients/group). Graphs show mean ± SEM; each point represents one mouse (B and C, E and F, I-K) or patient (L). B, E, F, I, J, K, and L: two-tailed unpaired Student’s *t* test; C: two-tailed Mann-Whitney test, and D, G, J (left): two-way ANOVA with Tukey’s or šídák’s multiple comparisons: ns = non-significant, *p < 0.05, **p < 0.01, ***p < 0.005, and ****p < 0.0001. See also Figure S1.

To investigate the impact of chronic stress on tumor growth, we employed the chronic unpredictable mild stress (CUMS) model in adult wild-type (WT) C57BL/6 mice (Figure 1A).^4^ At CUMS day 7, plasma corticosterone levels were significantly elevated in the CUMS group (Figure 1B). Using the elevated plus maze (EPM) to assess stress-related behavioral changes, we found that CUMS mice displayed a marked aversion to the open arm compared to Control mice, indicating increased anxiety (Figure 1C). CUMS did not alter the expression of corticosterone receptor NR3C1 across various splenic immune cell populations (Figure S1A). At CUMS day 7, colorectal cancer MC38 cells were injected subcutaneously into CUMS or control mice (Figure S1B); CUMS mice developed larger tumors than Control mice (Figure 1D). Flow cytometry analysis of intratumor immune cells showed no significant changes in innate or T cell subsets (Figures S1C and S1D); however, we found a significant reduction in intratumor germinal center (GC) B cells (B220^+^Fas^+^GL7^+^) as well as IgG^+^ B cells in CUMS mice at day 21 post tumor inoculation (Figure 1E). This was consistent with reduced plasma levels of anti-tumor IgG as compared to total IgG (Figures 1F and S1E). Interestingly, CUMS also increased intratumor corticosterone levels (Figure 1F). Furthermore, despite no change in total B cell numbers, the GC B cell response in the mesenteric lymph nodes (MLNs) and spleen was significantly diminished in CUMS tumor-bearing mice (Figure S1F), suggesting systemic B cell defects; consistent with this, intratumor TLS formation was diminished in CUMS mice (Figure S1G). Importantly, the depletion of B cells via anti-CD20 (Figure S1H) substantially abolished the effects of CUMS on tumor growth (Figure 1G); CUMS-driven acceleration of tumor growth was likewise abolished in IgG-deficient mice^22^ (Figure S1I), supporting a critical role for chronic stress-induced dysregulation of B cells in mediating the effect on tumor growth.

We performed single-cell RNA sequencing (sc-RNA-seq) of intratumor B cells isolated from Control and CUMS mice on day 21 post MC38 inoculation. Immunoglobulin heavy and light chain gene variants involved in B cell receptor (BCR) maturation, repertoire expansion, and antibody secretion, such as Ighv14-3, Ighv8-12, Igkv6-32, and Igkv1-110, were downregulated in intratumor B cells from CUMS mice (Figure 1H). Pathway enrichment analysis showed downregulation of BCR signaling, FcεR-mediated NF-κB signaling, and complement-mediated phagocytosis in intratumor B cells from CUMS mice, while suppressive TGF-β and SMAD-driven pathways were upregulated (Figure 1H). In addition, intratumor B cells from CUMS mice showed differential heavy and light chain gene usage in comparison to Control intratumor B cells (Figure S1J). In addition, using *Cdx2*-Cre^ERT^;*Apc*^fl/fl^ mice,^23^ a genetically driven orthotopic model of colorectal tumorigenesis, we found that CUMS similarly enhanced polyp formation, suppressed GC B cells, and increased corticosterone levels without any significant difference in the intratumor T cell compartment (Figures 1I and S1K).

To investigate whether the chronic stress-B cell circuit affects other types of tumors, CUMS or Control mice were inoculated subcutaneously with melanoma B16F10 cells. Similar effects of CUMS on tumor progression, intratumor GC B cells, and plasma anti-tumor IgG and intratumor corticosterone were observed in this model (Figures 1J and 1K, S1L, and S1M). Furthermore, we obtained human colorectal tumors and adjacent non-tumor colon tissues from 16 patients through the National Cancer Institute-supported Cooperative Human Tissue Network (NCI-CHTN) Program. We found increased corticosterone within tumor tissues relative to adjacent normal tissues from the same patients, suggesting an intratumor source of corticosterone. Importantly, we found fewer GC B cells in tumor tissues compared to adjacent colon tissues; the GC B cell response in these tissues negatively correlated with intratumor levels of corticosterone (Figure 1L). There were no significant differences in the responses of T cells or innate cells between tumor and adjacent tissues (Figure S1N). These data suggest a possible glucocorticoid-B cell circuit in human colon tumors.

### CUMS impairs antigen-specific B cell response in a gut microbiota-dependent manner

**Figure 2.**
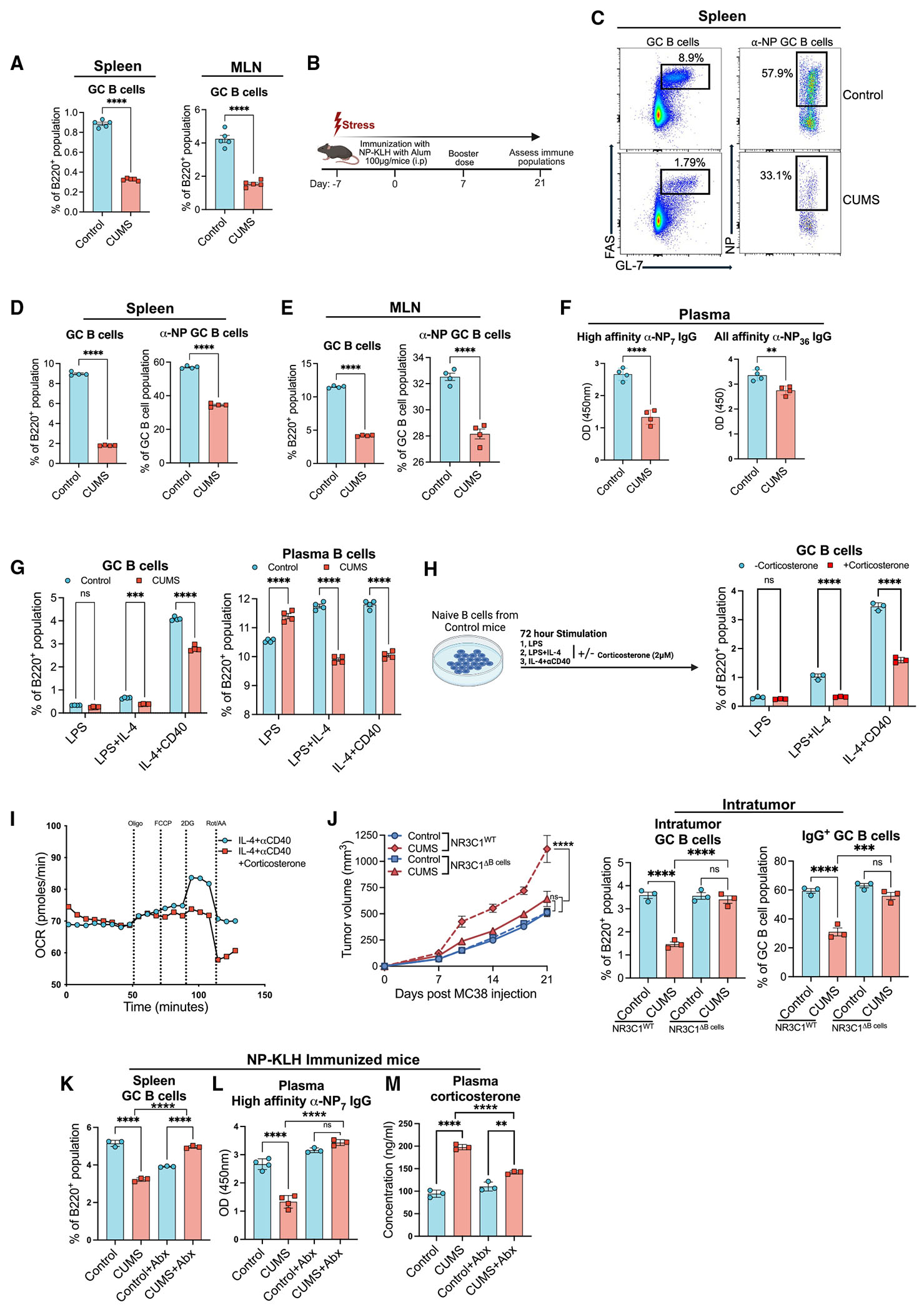
CUMS impairs antigen-specific B cell responses in a gut microbiota-dependent manner (A) Spleen and MLN GC B cells on CUMS day 21 (*n* = 5 mice/group). (B) NP-KLH immunization of Control or CUMS mice. (C-E) Splenic GC and NP-specific GC B cells on CUMS day 21 (*n* = 4 mice/group). (F) High-affinity anti-NP_7_ and all-affinity anti-NP_36_ plasma IgG (*n* = 4 mice/group). (G) GC and plasma B cell populations in immature splenic B cells from Control and CUMS mice, after stimulation *in vitro* with LPS, LPS+IL-4, or IL-4+αCD40 (*n* = 4 mice/group). (H) GC B cells in control immature splenic B cells stimulated *in vitro* with LPS, LPS+IL-4, or IL-4+αCD40 with or without corticosterone (*n* = 3 technical repeats/group). (I) Representative trace of OCR of three independent experiments for B cells stimulated in the presence and absence of corticosterone (*n* = 1 technical repeat). (J) MC38 growth kinetics intratumor GC and IgG^+^ GC B cell percentage in WT and B-cell-specific NR3C1-deficient mice (*Mb1*^Cre^*NR3C1*^fl/fl)^) (*n* = 3 mice/group). (K-M) Splenic GC B cells (K), high-affinity anti-NP plasma IgG (L), and plasma corticosterone (M) in antibiotic-treated control and CUMS mice following NP-KLH immunization (*n* = 3 mice/group). For A, D-H, and J-M, graphs show mean ± SEM; for A, D-F, and J-M, each point represents one mouse, while in G and H each point represents one technical replicate. A, D, E, F: two-tailed unpaired Student’s *t* test; G, H, J: two-way ANOVA with Tukey’s or šídák’s multiple comparisons, and J (right), K, L, M: one-way ANOVA with Tukey’s multiple comparisons: ns = non-significant, **p < 0.01, ***p < 0.005, and ****p < 0.0001. See also Figure S2.

At steady state, chronic stress significantly reduced GC B cells and plasma cells in the spleen and MLN (Figures 2A, S2A, and S2B). To determine if CUMS would impact antigen-specific GC responses, at CUMS day 7, WT mice were immunized intraperitoneally with B cell immunogen NP-KLH (4-Hydroxy-3-nitrophenylacetyl hapten conjugated to Keyhole Limpet Hemocyanin) with a booster dose 7 days later (Figure 2B). After two weeks (CUMS day 21), we found significantly fewer overall GC B cells and NP-specific GC B cells in the spleen and MLN of CUMS mice (Figures 2C–2E). CUMS mice had significantly lower levels of plasma high-affinity anti-NP_7_ IgG but only a modest reduction in plasma all-affinity anti-NP_36_ IgG (Figure 2F), suggesting defects in affinity maturation. To determine whether this reflects a temporal delay rather than a sustained defect induced by CUMS, we immunized mice one day prior to the initiation of CUMS; this also selectively reduced high-affinity anti-NP responses, indicating that CUMS imposes an immediate suppressive effect on B cell affinity maturation (Figure S2C). These findings corroborate our sc-RNA-seq data on intratumor B cells (Figure 1H) showing weaker BCR signaling and lower expression of heavy and light variable gene variants that are involved in regulating affinity maturation and B cell selection.^24,25^

Antigen-specific GC B cell responses and affinity maturation require T follicular helper (Tfh) cells.^26–28^ However, we found no changes in Tfh (CD3^+^CD4^+^BCL6^+^CXCR5^+^) cells in the tumors, spleens, and MLNs between Control or CUMS tumor-bearing mice (Figure S2D). We isolated naive splenic B cells from Control or CUMS mice and cultured them in the presence of T cell-independent (LPS, LPS+IL-4) or T cell-dependent (IL-4+CD40) stimuli, and found that B cells from CUMS mice showed significant impairment in differentiation to GC B cells or plasma cells (Figure 2G). GC B cells exhibit a preference for oxidative phosphorylation (OXPHOS) over glycolysis^29–31^; by Seahorse assay, we observed a reduced basal level of oxygen consumption in naive splenic B cells from CUMS mice, compared to B cells from Control mice (Figure S2E), suggesting an impairment in B cell switch toward OXPHOS for optimal GC response in CUMS mice. Furthermore, naive splenic B cells stimulated *in vitro* in the presence of corticosterone showed reduced differentiation into GC B cells and failed to switch to OXPHOS upon the inhibition of glycolysis (Figures 2H and 2I, and S2F). These results suggest that corticosterone directly skews the metabolic state of B cells, which likely hampers the GC reaction upon antigen exposure. Lastly, to test whether corticosterone acts intrinsically on B cells *in vivo*, we examined B-cell-specific *Nr3c1* knockout mice (*Mb1*^Cre^*Nr3c1*^fl/fl^). Loss of *Nr3c1* in B cells attenuated CUMS-induced tumor growth, and restored intratumoral GC B cells and plasma anti-tumor IgG (Figures 2J, S2G, and S2H). These results suggest that direct glucocorticoid signaling to B cells intrinsically impairs intratumor GC B cell responses in CUMS-induced tumor-bearing mice.

The gut microbiota is closely involved in antigen-dependent GC B cell responses.^32–34^ To determine if the gut microbiota was required for stress-induced B cell dysregulation, CUMS and Control mice were treated with broad-spectrum antibiotics (Abx) (Figure S2I) and immunized with NP-KLH. Abx treatment reduced splenic GC B cells in Control mice but restored GC B cells and high-affinity anti-NP_7_ plasma IgG in immunized CUMS mice to the levels seen in Control mice (Figure 2K and 2L). Of note, CUMS induced only a modest increase in plasma corticosterone of Abx-treated Control and CUMS mice (Figure 2M); this suggests the gut microbiota is critical for stress-induced glucocorticoid production.

### CUMS enhances tumor growth by modulating intratumor bacteria

**Figure 3.**
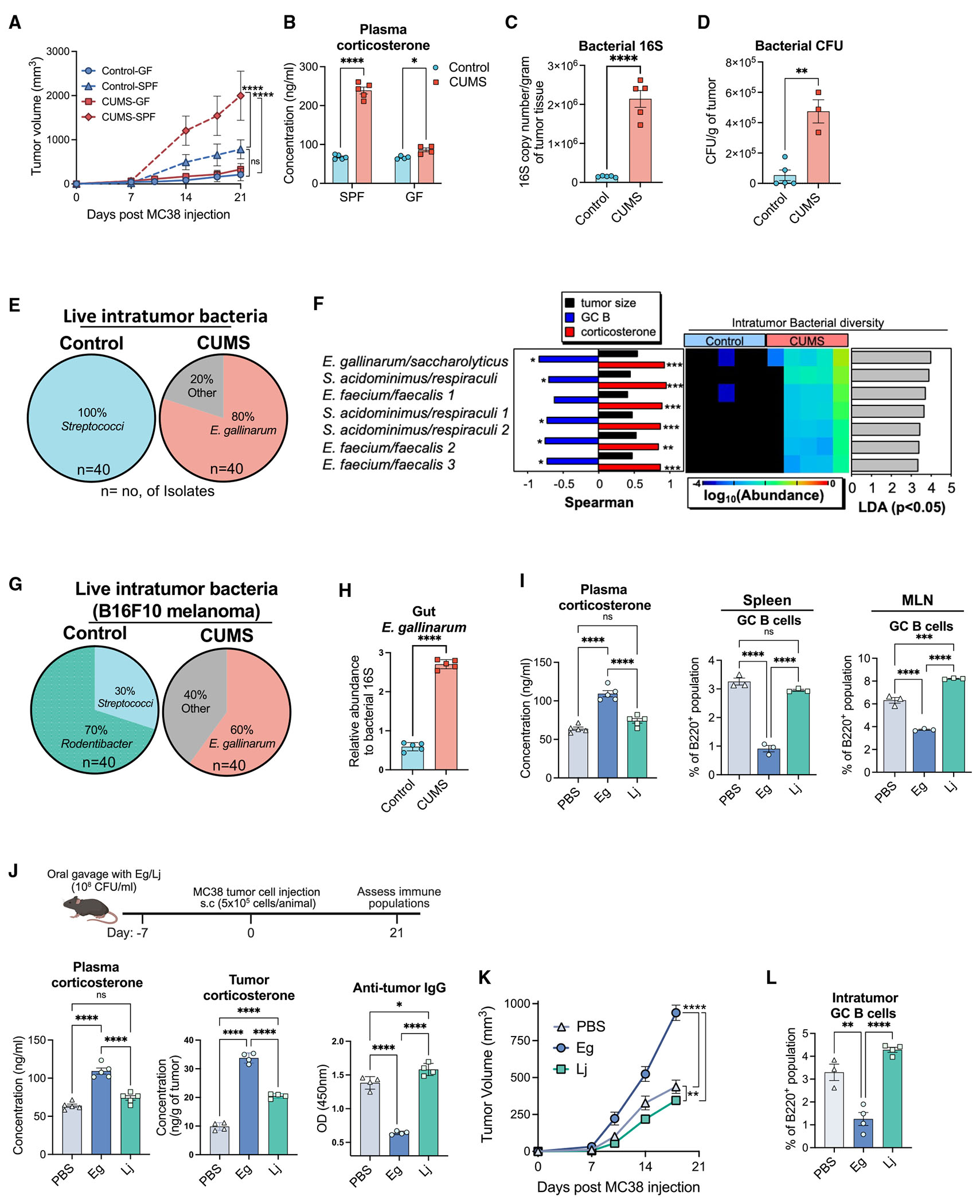
CUMS enhances tumor growth by modulating the intratumor microbiota (A) MC38 growth kinetics in Control and CUMS GF and SPF mice (*n* = 5 mice/group). At day 21, one mouse from the CUMS SPF group without signs of distress had tumor volumes exceeding 2000 mm^3^ and was euthanized immediately. (B) Plasma corticosterone at CUMS day 7 in SPF vs. GF mice (*n* = 5 mice/group). (C and D) Intratumor bacterial 16S DNA copy number (C) and tumor bacterial CFUs (D) from control and CUMS mice at day 21 post MC38 injection (n = 3–5 mice/group). (E) Identification of live bacteria cultured from tumors from Control and CUMS mice via Sanger sequencing of 16S rRNA (*n* = 40 isolates/group). (F) Spearman’s correlation of bacterial abundance with GC B cell abundance, corticosterone level, and tumor size in respective groups (left), LEfSe analysis showing bacterial OTUs that are differentially abundant between control and CUMS tumors (right) (n = 3–5 mice/group). (G) Identification of live bacteria cultured from tumors (21 days post B16F10 melanoma inoculation) from control and CUMS mice via Sanger sequencing of 16S rRNA (*n* = 40 isolates/group). (H) Relative abundance of *Enterococcus gallinarum* (Eg) in control and CUMS mice (*n* = 5 mice/group). (I) Plasma corticosterone (left), splenic and MLN GC B cells (middle/right) 7 days post Eg or *Lactobacillus johnsonii* (Lj) monocolonization in germ-free WT mice (n = 3–5 mice/group). (J) Plasma and tumor corticosterone (left/middle) and plasma anti-tumor IgG (right) in Control (PBS), Eg or Lj monocolonized mice 21 days post MC38 injection (n = 4–5 mice/group). (K and L) MC38 tumor growth (K) and intratumor GC B cells (L) in control (PBS), Eg- or Lj-monocolonized mice (n = 3–4 mice/group). For A-D and H-L, graphs show mean ± SEM; each point represents one mouse. Spearman’s rank correlation coefficient was used for correlation. C, D, H: two-tailed unpaired Student’s *t* test; E, G, I, J, L: one-way ANOVA with Tukey’s multiple comparisons and A, B, K: two-way ANOVA with Tukey’s multiple comparisons: ns = non-significant, *p < 0.05, **p < 0.01, ***p < 0.005, and ****p < 0.0001. See also Figure S3 and S4.

Germ-free (GF) control or CUMS mice exhibited similar MC38 growth kinetics (Figure 3A), and similar levels of intratumor GC B cells, innate immune cells, and T cell populations (Figures S3A–S3C), confirming that the gut microbiota mediated the effects of CUMS on B cells and anti-tumor immunity. Interestingly, CUMS only modestly increased plasma corticosterone in GF mice, which was much lower than that in SPF CUMS mice (Figure 3B). CUMS induced no significant changes in overall gut microbiota diversity, with very few taxa only modestly altered in CUMS mice (Figures S3D and S3E). Intratumor bacteria have been implicated in different types of tumors.^35–39^ We found increased bacterial 16S rRNA (Figure 3C) and live bacterial colonies (Figure 3D) in MC38 tumors from CUMS mice; 80% of the bacterial isolates from tumors in CUMS mice were *Enterococcus gallinarum* (Eg), while bacterial isolates from tumors in Control mice were predominantly *Streptococcus* (Figure 3E). 16S rRNA-sequencing of intratumor bacterial DNA consistently demonstrated increased abundance of Eg in tumors from CUMS mice, which negatively correlated with intratumor GC B cells and positively correlated with tumor size and intratumor corticosterone levels (Figure 3F). Interestingly, Eg also accounted for ~60% of live bacteria recovered from B16F10 melanoma tumors from CUMS mice; *Rodentibacter* and *Streptococci* were the dominant bacteria present in melanoma tumors from Control mice (Figures 3G, S3F, and S3G). We also found higher amounts of Eg 16S rRNA in fecal pellets from CUMS mice relative to Control mice (Figure 3H), and higher 16S rRNA copy numbers in polyps from CUMS-induced *Cdx2*-Cre^ERT^; *Apc*^fl/fl^ mice (Figure S3H), which also showed higher abundance of Eg isolates (Figure S3I).

To test whether Eg would promote tumor progression, we monocolonized GF WT mice with Eg or *Lactobacillus johnsonii* (Lj) as a control (Figure S3J). After 7 days, plasma corticosterone levels were significantly increased in Eg-monocolonized mice, compared to Lj-monocolonized mice (Figure 3I), despite similar gut colonization between Eg and Lj (Figure S3J). After 21 days, we found significantly fewer GC and plasma B cells in the spleens and MLNs of Eg-monocolonized mice (Figures 3I and S4A); there were no notable differences in other immune populations (Figures S4B and S4C).

Furthermore, in Eg-monocolonized mice inoculated with MC38 tumor cells, we found higher plasma and intratumor corticosterone levels and lower levels of plasma anti-tumor IgG (Figure 3J). Eg translocated to tumors more efficiently than Lj (Figure S4D). Importantly, Eg-monocolonized mice showed larger tumors and reduced intratumor GC B cells, plasma cells, and IgG^+^ B cells (Figures 3K, 3L, and S4E). These results, mirroring those found in CUMS tumor-bearing mice, suggest that Eg mediated the effects of CUMS on anti-tumor B cell response and tumor progression.

### Eg DNA induces corticosterone production by intratumor fibroblasts via TLR9

**Figure 4.**
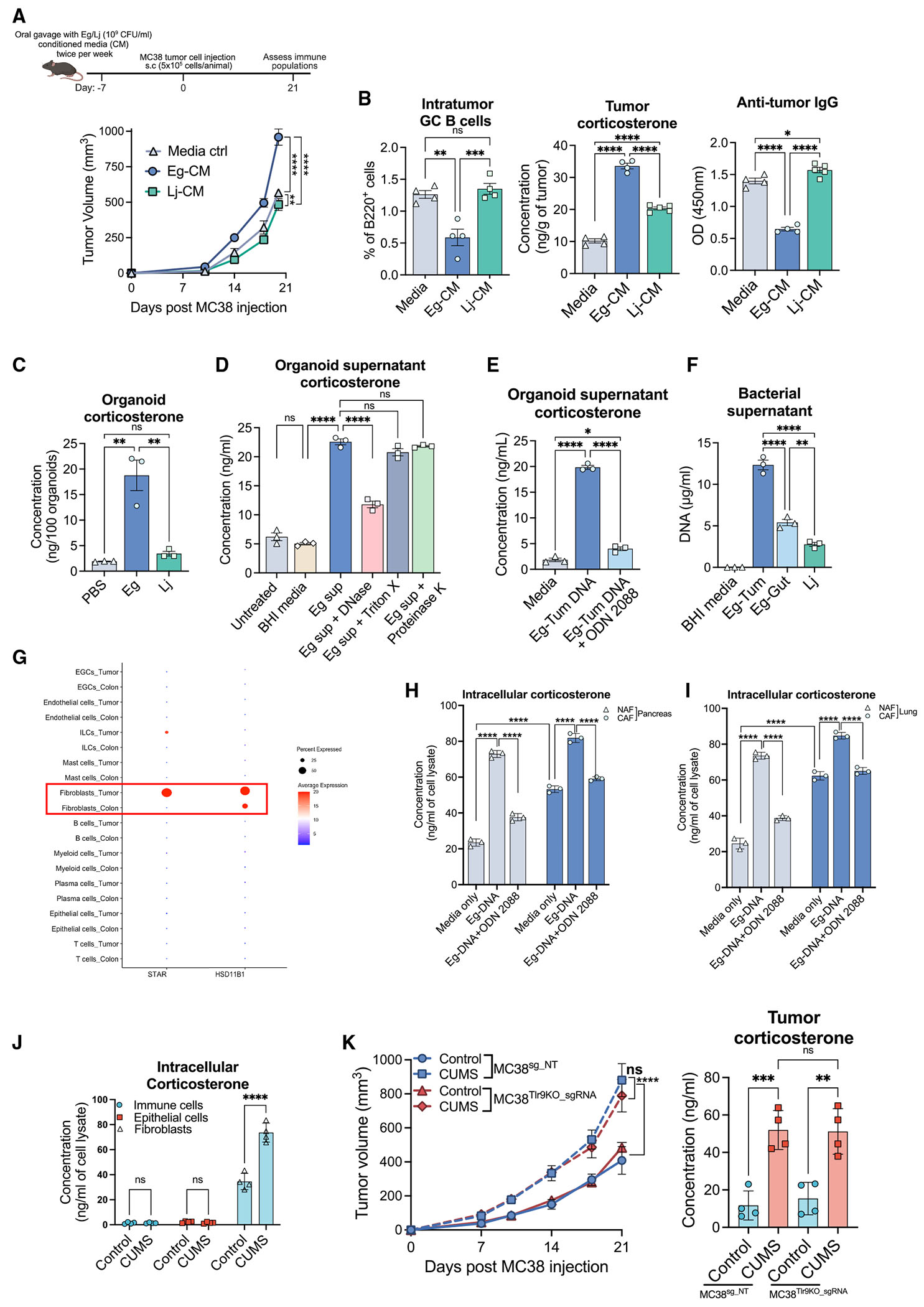
Eg DNA induces corticosterone production by cancer-associated fibroblasts (CAFs) via TLR9 (A) MC38 growth kinetics and (B) intratumor GC B cells (left), tumor corticosterone (middle), and plasma anti-tumor IgG (right) in GF mice orally gavaged every other day with BHI (control), Eg-conditioned, or Lj-conditioned media (n = 4–5 mice/group). (C) Corticosterone production by organoids generated from intestinal crypts of Eg and Lj-gavaged mice (*n* = 3 mice/group). (D) Corticosterone production by intestinal organoids treated with BHI media (control), Eg culture supernatant, or Eg culture supernatant pretreated with DNase0, Triton X, or proteinase K (*n* = 3 technical repeats/group). (E) Corticosterone production by organoids treated with Eg-Tum DNA in the presence or absence of TLR9 antagonist ODN2088 (*n* = 3 technical repeats/group). (F) DNA concentrations in culture supernatant of tumor-derived Eg (Eg-Tum), gut-derived Eg (Eg-Gut), and Lj (*n* = 3 technical repeats/group). (G) Expression of extra-adrenal corticosterone synthesis genes in human colon tissues and colon tumors, from publicly available sc-RNA-seq data (GSE236384) with color and size of circle depicting average expression and percent expressed, respectively (*n* = 17 patients/group). (H and I) Intracellular corticosterone in human lung and pancreatic NAFs or cancer-associated fibroblasts (CAFs) stimulated with Eg-DNA in the presence or absence of ODN2088 (*n* = 3 technical repeats/group). (J) Intracellular corticosterone in FACS sorted epithelial (EpCAM^+^CD31^−^CD45^−^), immune (CD45^+^), and CAF (PDGFR^+^PDPN^+^CD45^−^CD31^−^) cells from MC38 tumors (*n* = 4 mice/group). (K) Tumor growth (*n* = 4) and intratumor corticosterone levels in mice inoculated with control MC38 (sg-NT) or TLR9-deficient MC38 (Tlr9-sgRNA) cells (*n* = 4 mice/group). Graphs show mean ± SEM; for B-C and J-K, each point represents one mouse (n = 3–5), while for D-F each point represents one technical replicate, and for H-I each point represents one technical repeat. B, C, D, E, F, and K (right): one-way ANOVA with Tukey’s multiple comparisons; A, H, I, J, and K (left): two-way ANOVA with Tukey’s multiple comparisons: ns = non-significant, *p < 0.05, **p < 0.01, ***p < 0.005, and ****p < 0.0001. See also Figures S4 and S5.

To determine how Eg modulated anti-tumor B cell response and tumor progression, we orally gavaged GF mice with Eg (Eg-CM) or Lj (Lj-CM) conditioned culture media twice a week, and injected MC38 tumor cells at day 14 following the first gavage of CM (Figure 4A). Eg-CM mice exhibited changes in tumor progression, intratumor GC B cells, tumor corticosterone levels, and plasma anti-tumor IgG akin to Eg-monocolonized tumor-bearing mice (Figures 4A and 4B). These phenotypes were also observed following intratumoral injection of Eg-CM, including increased tumor growth and GC B cell impairment (Figure S4F). These results suggest Eg-derived factors promote a tumor-permissive microenvironment.

Reanalysis of publicly available human colorectal sc-RNA-seq data (GSE236384)^40^ showed that, compared to normal adjacent tissues, colon tumor tissues showed increased expression of genes involved in corticosterone and cholesterol biosynthesis, including *StAR*, *STARD4*, *HSD17B6*, *CYP39A1*, *SMO,* and *DHCR7* in both fibroblasts and epithelial cells (Figure S5A). To determine whether Eg could induce glucocorticoids, we generated organoids from intestinal crypts of Eg- and Lj-colonized mice; higher corticosterone levels (Figure 4C) and increased expression of enzymes involved in extra-adrenal corticosterone pathways (*StAR*, *Cyp11a1*, *Cyp11b1,* and *Hsd11b2*) (Figure S5B) were detected in organoids derived from Eg-monocolonized mice. Using an NR3C1 reporter cell line to detect corticosterone, we detected no functional corticosterone in Eg culture supernatants (Figures S5C and S5D), ruling out the possibility of corticosterone production by Eg.

To identify the secretory factor(s) from Eg that contributed to tumor progression, we cultured intestinal organoids from WT SPF mice with Eg culture supernatants (EgSup), which had been pretreated with DNase0 to degrade bacterial DNA, Triton X- to disrupt lipids, or proteinase K to degrade proteins. DNase0 pretreatment significantly decreased organoid corticosterone production (Figure 4D), suggesting that corticosterone production is likely induced by Eg-secreted DNA. Bacterial DNA is recognized by TLR9 on intestinal epithelial cells^41,42^; we found that the TLR9 antagonist ODN2088 blocked corticosterone production in Eg DNA-treated intestinal organoids (Figure 4E), suggesting that Eg-secreted DNA induced corticosterone production via TLR9.

Interestingly, we found that Eg isolated from tumors (Eg-Tum) secreted more DNA than either Lj or a gut isolate of Eg (Eg-Gut) (Figure 4F). To investigate the intratumor source of glucocorticoids induced by Eg-Tum DNA that suppressed GC B cells in CUMS mice, we reanalyzed the publicly available scRNA-sequencing data (GSE236384) on human colon tissues and tumors. Fibroblasts exhibited the highest expression of extra-adrenal glucocorticoid synthesis genes, such as *StAR*, *Hsd11b1,* and *Cyp11a* (Figures 4G and S5E), which were upregulated further in cancer-associated fibroblasts (CAFs). Increasing evidence suggests CAFs play a crucial role in remodeling tumor microenvironments, influencing tumor growth, metastasis, and drug resistance^43^; TLR9 signaling has been implicated in CAF activation and tumor invasiveness.^44,45^ Using NAF (normal fibroblasts) and CAF cell lines derived from human patients with lung or pancreatic cancer, we showed that Eg-DNA stimulated NAFs and CAFs to produce corticosterone via TLR9; baseline intracellular corticosterone was also higher in CAFs (Figures 4H and 4I), which is consistent with increased expression of *StAR* and *hsd11b1* in CAFs compared to NAFs from scRNA-seq data on colon tumors (Figure 4G). To directly assess the cellular source of corticosterone within tumors, we sorted epithelial, immune cells, and CAFs from MC38 tumors and measured intracellular corticosterone. Notably, corticosterone was detectable only in CAFs, and was significantly higher in CAFs from CUMS mice (Figure 4J), suggesting CAFs as the dominant intratumoral source of corticosterone. We also observed similar tumor growth and intratumor corticosterone levels in mice inoculated with control MC38 (sg-NT) or TLR9-deficient MC38 cells (Figures 4K and S5F). These data show that CAFs provide a critical source of intratumor glucocorticoids via the Eg DNA-TLR9 circuit in response to chronic stress.

### Phage DNA released by tumor-associated bacteria induces corticosterone production by cancer-associated fibroblasts

**Figure 5.**
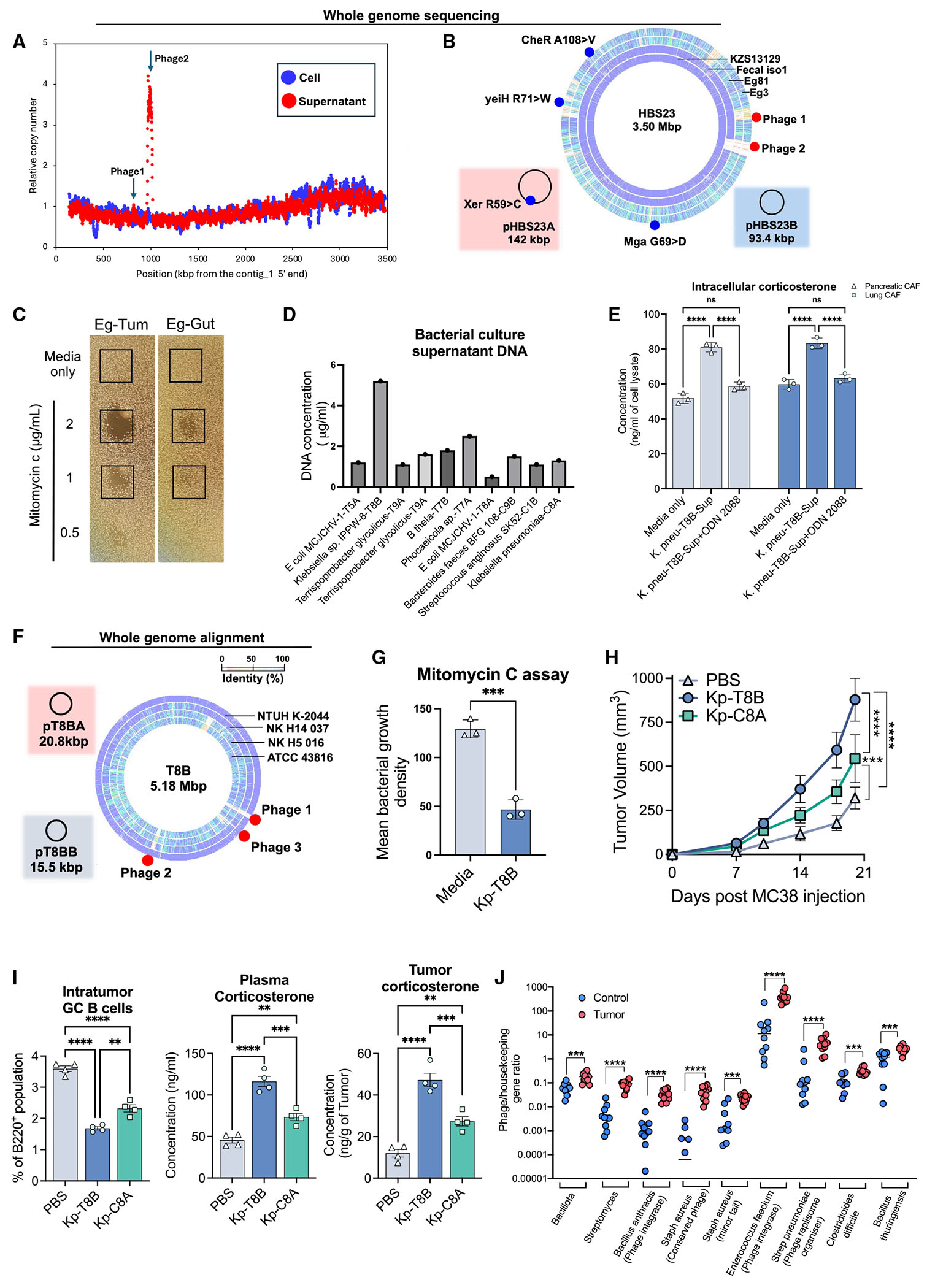
Phage DNA released by murine and human tumor-associated bacteria induces corticosterone production by cancer-associated fibroblasts (A) Whole-genome sequencing of DNA isolated from Eg-Tum cells or culture supernatant; phage gene copy number shown as phage gene copies per million Eg genes. (B) Alignment of whole-genome sequences of Eg-Tum (HBS23) with Eg-Gut (KZS13129) and published Eg strains (Fecal iso 1, Eg3, and Eg81); phages 1 and 2 and selected mutations are indicated. (C) Phage induction by mitomycin C treatment of lawn colonies of Eg-Tum and Eg-Gut. (D) Supernatant DNA quantification from cultures of bacteria isolated from fresh human colorectal tumors (*n* = 1 isolate/group). (E) Intracellular corticosterone in human lung and pancreatic cancer-associated fibroblasts (CAFs) stimulated with *Klebsiella pneumoniae*-T8B (Kp-T8B) supernatant in the presence or absence of ODN2088 (*n* = 3 technical repeats/group). (F) Alignment of whole-genome map of Kp-T8B with publicly available *K. pneumoniae* strains NK_H5_016, NK_H14_037, NTUH K-2044, and ATCC 43816, with phages 1–3 indicated. (G) Quantification of phage-mediated lysis of lawn colonies (*n* = 3 technical repeats/group). (H) MC38 growth kinetics (*n* = 4) and (I) intratumor GC B cells, plasma, and tumor corticosterone in mice orally gavaged twice a week with PBS (control), Kp-T8B, or Kp-C8A (*n* = 4 mice/group). (J) Reanalysis of publicly available glioma and brain metastasis datasets. Ratios of phage-derived reads in tumor samples relative to oral controls were shown in specific phage-associated bacterial hosts. Samples with more than 100000 phage reads (*n* = 10 or 11 for control or tumor group patients, respectively) are shown. For E and G–J, graphs show mean ± SEM; for E and G, each point represents one technical replicate, for I, each point represents one mouse, and for J, each dot represents one patient. A: Mann-Whitney test; G, J: two-tailed unpaired Student’s *t* test; I: one-way ANOVA with Tukey’s multiple comparisons, and E, H: two-way ANOVA with Tukey’s multiple comparisons: ns = non-significant, **p < 0.01, ***p < 0.005, and ****p < 0.0001. See also Figures S6 and S7.

We performed whole-genome sequencing of Eg-Tum intracellular DNA (Eg-cell) and secreted DNA (Eg-Sup), with Eg-Gut as a control. We found that two prophages, noted as Phage 1 and Phage 2, were integrated within the genomes of both Eg-Tum and Eg-Gut. Surprisingly, we found significantly increased copy numbers of Phage 2 in Eg-Sup, compared to the whole-cell genome of Eg-cell (Figure 5A), suggesting that release of Phage 2 might explain the increased amounts of DNA from the supernatant of Eg-Tum. Phylogenetic analysis of >200 published Eg isolates showed Eg-Tum (HBS123) was most closely related to Eg-Gut (KZS13129), suggesting Eg-Tum likely diverged from Eg-Gut through mutations to adapt to the tumor environment (Figure S6). Using Eg-Gut and other publicly available Eg strains (Fecal iso 1, Eg3, and Eg83) as references, we found 3 mutations (CheR A108 > V, yeiH R71 > W and Mga G69 > D) in Eg-Tum (HBS123) (Figure 5B). Mga, a transcription factor, plays a crucial role in bacterial virulence, adherence, and evasion of host immune response.^46–48^ CheR, a methyltransferase, is essential for chemotaxis, adhesion, and virulence.^49–51^ YeiH, a transmembrane protein, remains relatively understudied; however, some reports suggest involvement in chemotaxis and induction of pathogenic CD8^+^ T cells in autoimmune conditions.^52^ Thus, it is plausible that mutations in these genes allowed Eg-Tum to translocate to tumors and survive within the tumor microenvironment.

The release of phage 2 from Eg-Tum suggests phage 2 may become lytic within the tumors of CUMS mice. To test this, we added mitomycin C, which induces prophages to become lytic, to lawns of Eg-Tum or Eg-Gut. A low concentration of mitomycin C was sufficient to induce apparent plaque formation, indicative of phage lysis, with Eg-Tum but not Eg-Gut (Figure 5C), suggesting that, even though both Eg-Tum and Eg-Gut harbor prophages 1 and 2 in their genomes, the repression of prophage 2 gene expression was weaker in Eg-Tum, likely due to the additional mutations in Eg-Tum (Figure 5B). Mutations in chemotaxis-associated genes (*Mga*, *CheR*, *YeiH*) may alter motility,^53,54^ which may lead to SOS activation and phage induction, as RecA, a key regulator of the lytic cycle,^55^ interacts with chemotaxis-related quorum-sensing molecules CheW and CheA.^56,57^ These mutations, along with phage 2 activation, in Eg-Tum might be a necessary adaptation to enable Eg-Tum to traffic from the gut to tumors and survive within the tumor microenvironment.

To validate phage activation and DNA-induction of corticosterone by CAFs in human tumors, we isolated live bacteria from fresh human colorectal tumors obtained through the NCI-CHTN program. Quantification of DNA in culture supernatants of >10 bacterial isolates showed significantly higher amounts of extracellular DNA in *Klebsiella pneumoniae*-T8B (Kp-T8B) (Figure 5D). Treating CAFs with culture supernatants from Kp-T8B increased corticosterone production, which was abolished in the presence of ODN2088 (Figure 5E). We performed whole-genome sequencing of Kp-T8B and aligned it to publicly available *K*. *pneumoniae* strains, including NK_H5_016, NK_H14_037, NTUH K-2044, and ATCC 43816. We identified phages (phages 1, 2, and 3) in Kp-T8B only (Figure 5F) and found that a low dose of mitomycin C induced clear plaque formation in Kp-T8B lawns (Figures 5G and S7A).

To functionally confirm the role of Kp-T8B *in vivo*, we orally gavaged mice with KpT8B or Kp-C8A, a *K. pneumoniae* isolate with minimal DNA release, which was isolated from an adjacent normal colon tissue (Figure 5D), followed by MC38 inoculation 7 days after the first gavage (Figure S7B). Notably, Kp-T8B, but not Kp-C8A, significantly accelerated tumor growth, increased plasma, and intratumoral corticosterone, and reduced intratumoral GC B cells (Figures 5H and 5I). Notably, Kp-T8B and Eg-Tum share key functional features, such as conserved virulence-associated genes, *ClpP*, *lap* and *sigA*,^58^ despite taxonomic differences (Figure S7C). To determine whether intratumor phages are present in tumors remote from the gut, we analyzed publicly available metagenomic data (PRJNA1023304)^59^ from glioma and metastatic brain tumors. We detected increased phage genes per housekeeping gene homolog ratio of specific individual taxa in tumor tissue, relative to oral microbiome controls, indicating phage enrichment in non-GI cancers. Of note, the proteins of the taxon that shows the highest ratio with a statistically significant difference between tumors and control samples were binned to *Enterococcus faecium* integrase by homology (Figure 5J). Notably, the original study^59^ reported enrichment of TLR9 and downstream signaling in regions of brain metastatic tumors with high microbial DNA levels, supporting the notion that phage DNA engages TLR9 within non-GI tumors as well.

### Direct corticosterone signaling to intestinal epithelial cells impairs gut barrier and allows Eg translocation to tumors

**Figure 6.**
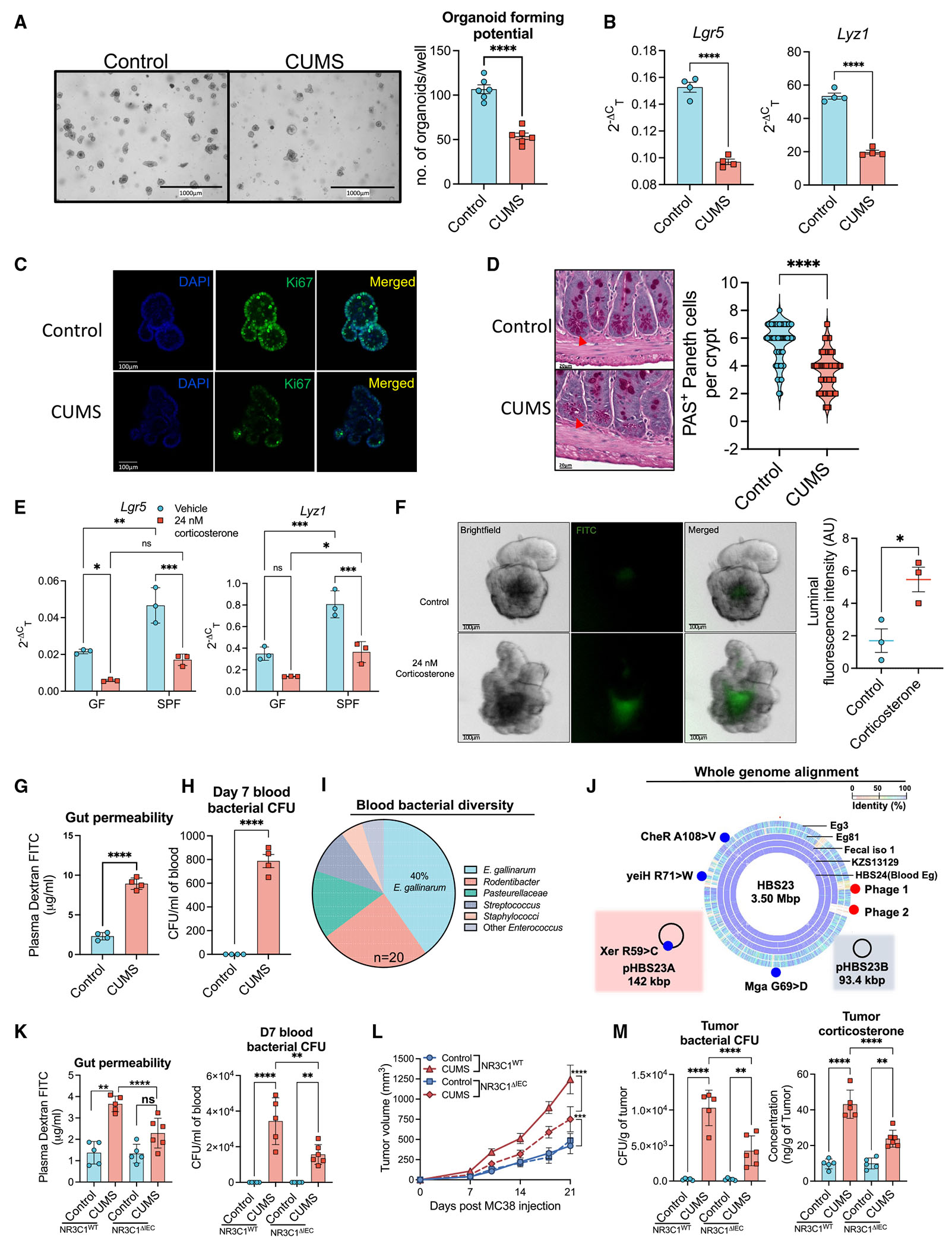
Direct corticosterone signaling to intestinal epithelial cells impairs gut barrier and allows translocation of adapted Eg to tumors (A) Organoid-forming potential of intestinal crypts from control and CUMS mice (*n* = 6 mice/group). (B) *Lgr5* and *Lyz1* expression in control and CUMS intestinal organoids; gene expression was quantified by qRT-PCR using the comparative Ct method. Ct values of target genes were first normalized to the housekeeping gene *GAPDH* for each sample by calculating ΔCt values (*n* = 4 mice/group). (C) Control and CUMS intestinal organoids stained for Ki67. (D) Representative images and quantification of PAS^+^ Paneth cells (highlighted by arrow heads) at the base of the intestinal crypts in day 7 control and CUMS mice (*n* = 3 mice/group). (E) *Lgr5* and *Lyz1* expression in intestinal organoids derived from naive GF or SPF mice and treated with corticosterone (*n* = 3 mice/group). (F) *In vitro* FITC-dextran permeability in colon organoids (*n* = 3 mice/group). (G) Plasma FITC-dextran levels at CUMS day 7 (*n* = 4 mice/group). (H) CFUs of live bacteria cultured from the blood of control or CUMS mice (*n* = 4 mice/group), and (I) relative abundance of different bacterial species among 20 isolates sequenced (*n* = 20 isolates/group). (J) Alignment of whole-genome sequences of a blood Eg isolate from a day 7 CUMS mouse (HBS24) with Eg-Tum (HBS23), Eg-Gut (KZS13129), and published Eg strains (Fecal iso 1, Eg3, and Eg81); Phages 1 and 2 and selected mutations are indicated. (K-M) Gut permeability and blood bacterial CFUs at CUMS day 7 (n = 5–6 mice/group) (K), MC38 growth kinetics (n = 5–6 mice/group) (L), and day 21 intratumor bacterial CFUs and corticosterone (M) in NR3C1^WT^ and intestinal epithelium-specific NR3C1 deficient mice (NR3C1^IECs^) (n = 5–6 mice/group). Graphs show mean ± SEM; for A-B, E-H, K, and M, each point represents one mouse. A, B, F, G, and H: two-tailed unpaired Student’s *t* test; D: two-tailed unpaired *t* test with Welch’s correction; I, K, and M: one-way ANOVA with Tukey’s multiple comparisons and E, L: two-way ANOVA with Tukey’s multiple comparisons: ns = non-significant, *p < 0.05, **p < 0.01, ***p < 0.005, and ****p < 0.0001. See also Figures S7 and S8.

To investigate how gut-derived Eg gains access to tumors under chronic stress conditions, we isolated intestinal crypts from both the SI and the colon of day-7 Control and CUMS mice. The intestinal crypt stem cells from both colon (Figure 6A) and SI (Figure S7D) of CUMS mice displayed a significant reduction in organoid-forming potential compared to those of Control mice, indicating impaired proliferation and differentiation of intestinal stem cells. Intestinal stem cell marker *Lgr5* and Paneth cell marker *Lyz1* were significantly downregulated in organoids derived from CUMS mice (Figure 6B); Ki67 immunostaining was also reduced, suggesting reduced proliferative potential (Figure 6C). This was validated by Periodic Acid-Schiff staining (Figure 6D) and immunohistochemistry labeling of LYZ1 (Figure S7E), which highlighted a significant reduction in Paneth cell abundance at the base of intestinal crypts of CUMS mice. These data suggest a possible loss of critical signals for stem cell proliferation and maintenance, as well as impaired mucosal defense in CUMS mice. Intestinal organoids from GF mice expressed less *Lgr5* and *Lyz1* than organoids derived from SPF mice (Figure 6E); importantly, corticosterone treatment further reduced expression of both genes in both GF and SPF-derived organoids, indicating a synergistic effect of microbial deprivation and corticosterone signaling on intestinal stem cell and Paneth cell function.

The reduction in stem and Paneth cells in CUMS intestines suggests impairments in epithelial regeneration and mucosal defense, likely due in part to direct corticosterone signaling. This is supported by increased FITC-Dextran permeability in corticosterone-treated colon organoids (Figure 6F), which also showed downregulation of key tight junction genes such as *Ocln*, *Cldn1,* and *Tjp1* (Figure S7F). These data show that corticosterone directly impacts epithelial barrier integrity, which could allow Eg to translocate from the gut to tumors. Day-7 CUMS mice also exhibited greater intestinal permeability, as measured by FITC-Dextran (Figure 6G), and we detected increased numbers of live bacteria in the bloodstream at CUMS day 7 (Figure 6H); Eg accounted for approximately 40% of all bacterial isolates sequenced (Figure 6I). Whole-genome sequencing of blood Eg isolates confirmed that they harbored the same mutations found in tumor-resident Eg-Tum (HBS23), as well as prophages 1 and 2 (Figure 6J), suggesting that CUMS-induced gut permeability allows adapted Eg within the gut, which possesses increased virulence against the host and propensity for phage activation, to translocate to tumors. Importantly, we found that specific deletion of *NR3C1* in intestinal epithelial cells in *NR3C1*^ΔIECs^ mice significantly reduced gut permeability following CUMS; CUMS tumor-bearing *NR3C1*^ΔIECs^ mice exhibited decreased bacterial translocation to the blood and tumors, as well as a concomitant reduction in plasma and intratumoral corticosterone. Importantly, epithelial cell-specific *NR3C1* deletion also attenuated CUMS-induced tumor growth, demonstrating a direct causal role for corticosterone-NR3C1 signaling in intestinal epithelial cells promoting microbial translocation and downstream tumor progression (Figures 6K–6M, S7G, and S7H).

Despite transient gut permeability and presence of Eg in the blood of CUMS mice, these mice exhibited no changes in IFNγ^+^CD4^+^ or CD8^+^ T cells, and Th17 cells (Figure S8A). These data argue against a sepsis-like inflammatory state, likely due to the suppressive effects of glucocorticoids. MC38-inoculated mice treated with anti-PD-1 showed an increase in circulating bacterial CFUs, consistent with prior reports^60,61^; however, this did not affect intratumor bacterial burdens (Figure S8B). This may be in part due to the anti-PD-1-mediated activation of myeloid cells^62^ that eradicate translocated bacteria in the blood. Furthermore, mice subjected to chronic low-grade gut injury (0.2% DSS for 21 days) had reduced intratumoral GC B cells and increased tumor growth (Figures S8C and S8D), likely attributable to elevated intratumoral corticosterone (Figure S8E); this was not observed in mice subjected to acute gut injury (2% DSS for 7 days). This suggests that chronic low-grade gut injury mimics chronic stress. These findings reveal a gut-tumor axis driven by chronic stress that permits the escape of adapted Eg from the gut due to corticosterone signaling-mediated disruption of intestinal stem cells and Paneth cells and transient loss of gut barrier integrity; within the tumor microenvironment, Eg-derived phage DNA induces intratumor corticosterone in fibroblasts via TLR9 to dampen anti-tumor B cell responses.

### Targeting intratumor TLR9 or Eg reverses the effect of CUMS on tumor growth

**Figure 7.**
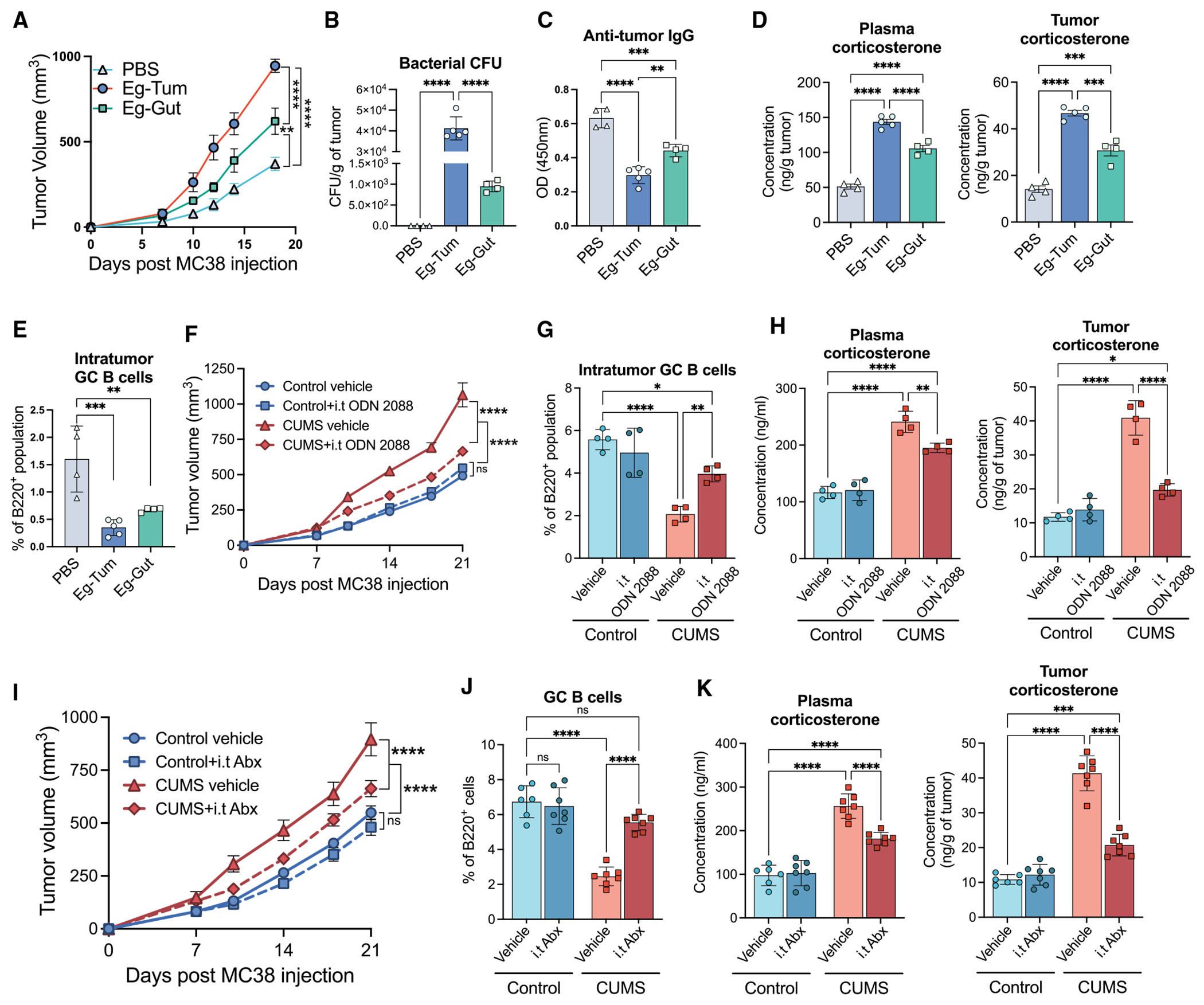
Targeting intratumor TLR9 or Eg reverses the effect of CUMS on tumor growth (A-E) WT SPF mice were orally gavaged with Eg-Tum or Eg-Gut twice a week; MC38 tumor cells were injected s.c. 1 day post first gavage. (A) MC38 growth kinetics (n = 4–5 mice/group), (B) intratumor bacterial CFUs, (C) plasma anti-tumor IgG, (D) plasma and intratumor corticosterone, and (E) intratumor GC B cells (n = 4–5 mice/group). (F-H) MC38 tumors were injected with ODN2088 or vehicle, every other day starting at day 7 post tumor inoculation; (F) MC38 growth kinetics (*n* = 4 mice/group), (G) intratumor GC B cells, and (H) plasma and intratumor corticosterone (*n* = 4 mice/group). (I-K) MC38-inoculated mice were treated with ampicillin via intratumor injection. (I) MC38 growth kinetics. (J) intratumor GC B cells and (K) plasma and intratumor corticosterone (n = 6–7 mice/group). Graphs show mean ± SEM; each point represents one mouse. B, C, D, E, G, H, J, and K: one-way ANOVA with Tukey’s multiple comparisons and A, F, I: two-way ANOVA with Tukey’s multiple comparisons: ns = non-significant, *p < 0.05, **p < 0.01, ***p < 0.005, and ****p < 0.0001. See also Figure S8.

To explore whether increased phage abundance accelerated tumor growth, we orally gavaged SPF mice with Eg-Tum or Eg-Gut the day before MC38 tumor inoculation and twice a week thereafter (Figure S8F). Mice given Eg-Tum exhibited faster tumor growth and greater live intratumor bacteria than mice given Eg-Gut (Figures 7A and 7B), as well as reduced plasma anti-tumor IgG, higher plasma and intratumor corticosterone levels, and decreased intratumor and splenic GC B cells (Figures 7C–7E, S8G).

Since TLR9 inhibition blocked corticosterone production in Eg DNA-treated intestinal organoids and human fibroblasts (Figures 4E, 4H, and 4I), we investigated whether disengaging the Eg phage DNA-CAF circuit could reverse the effect of CUMS on tumor growth. Intratumoral injection of ODN2088 substantially reduced tumor growth and enhanced intratumor GC B cell numbers in CUMS mice, without impacting total B cell abundance (Figures 7F–7H, S8H, and S8I), and reduced plasma and intratumor corticosterone (Figure 7H). Furthermore, local intratumor injection of Eg-DNA showed the same pattern of increased tumor growth and reduced intratumor GC B cells (Figure S8J). Finally, to investigate if localized targeting of intratumor Eg would block the effects of CUMS on tumor progression, we first determined that Eg-Tum was highly sensitive to ampicillin *in vitro* (not shown) and then injected ampicillin directly into tumors in Control and CUMS mice every other day starting on day 7 following MC38 inoculation. Intratumor ampicillin significantly reduced tumor volume, improved intratumor GC B cell numbers, and reduced intratumor corticosterone in CUMS but not Control mice (Figures 7I–7K). Together, these results suggest that targeting Eg or blocking the phage-TLR9 circuit within tumors could reduce intratumor corticosterone and mitigate the effects of chronic stress on anti-tumor B cell response and tumor progression.

## DISCUSSION

Chronic stress is a driver of tumor progression.^4,63–66^ In this study, we have demonstrated an important *trans*-kingdom circuit of intratumor gut-originating bacteria, phages, and B cells that is unleashed by chronic stress to promote tumor progression. Importantly, we show that chronic stress-induced gut barrier dysfunction is mediated by NR3C1 signaling in intestinal epithelial cells, leading to increased gut permeability and the selection and translocation of adapted gut Eg, which accumulates in tumors, where phage DNA from Eg induces local corticosterone via TLR9 in CAFs, thereby dampening the intratumor B cell response. Therapeutic strategies to disengage this circuit in patients with cancer could potentiate anti-tumor immunity and improve immunotherapy responses.

In mice, prednisone, dexamethasone, or corticosterone treatments reduced bone marrow B cells and B cell progenitors.^67–69^ Our data demonstrate a direct, cell-intrinsic role for corticosterone in reducing B cell metabolic flexibility via the GR. Chronic stress-induced suppression of GC B cell activity may reflect a physiological re-prioritization toward immediate survival over investment in long-lasting immunity. In contrast, GC B cells respond to acute stressors, such as infection or immunization, via stress-induced β2-adrenergic receptor (β2AR) activation on B cells, which favors affinity maturation and generation of high-affinity IgG at the expense of B cell clonal expansion.^70^ In addition, given our observation that corticosterone disrupts gut epithelial barrier integrity, restricting antigen-specific B cell activation through sustained corticosterone-NR3C1 signaling may represent an advantageous physiological mechanism to limit the risk of autoimmune responses.

Intratumor bacteria have been found in diverse human cancers that may alter the intratumor microenvironment and immune response^12,71,72^; microbial DNA in metastatic brain tumors positively correlated with intratumor TLR9 expression and downstream signaling.^59^ Our reanalysis of this study’s data revealed increased phage enrichment in tumors and association of bacterial burden with TLR9 pathway activation, suggesting that the phage-TLR9 circuit may extend beyond gastrointestinal cancers. Notably, Eg has also been detected in liver biopsies from patients with autoimmune diseases, attributed to its ability to acquire mutations that enable translocation to the liver.^73,74^ Mutations acquired by tumor-derived Eg may facilitate the lytic reactivation of Phage 2, stimulating CAF glucocorticoid production via TLR9 sensing of phage DNA. CAFs help create a tumor microenvironment conducive to progression, metastasis, and drug resistance^75^; our results illuminate a mechanism whereby CAFs suppress intratumoral GC B cells by supplying a local source of glucocorticoids.

Bacteriophages are increasingly investigated as therapeutic agents to eliminate bacteria or tumor cells^76–78^; for example, prophage-encoded antigens from gut-associated *Enterococcus hirae* have been shown to enhance antitumor immunity through cross-reactive CD8^+^ T cell activation.^77^ However, our study now presents evidence that phages may also facilitate tumor progression by impairing anti-tumor B cell responses. Patients with cancer endure elevated levels of chronic stress, which may heighten the risk of phage activation from intratumoral bacteria, thereby impairing antitumor immunity and responses to immunotherapies. Therapeutics targeting the intratumoral phage-CAF axis could represent an effective approach to remodel the TME and augment antitumor immunity.

### Limitations of the study

While our preclinical mouse models provide mechanistic insights into chronic stress-induced tumor progression via the intratumor phage-CAF-B cell circuit, translation to human cancers requires further investigation due to interspecies differences in microbiota composition, immune responses, and stress physiology. Furthermore, the CUMS model, although validated for anxiety-like behaviors in mice, cannot fully recapitulate the complex psychosocial stressors experienced by human patients with cancer. Additionally, although our data on human tumors show an inverse correlation between intratumor corticosterone and GC B cells in colorectal tumors, and the presence of phages in glioma and brain tumors, future longitudinal or interventional studies are needed to establish causality. In our study, we focused on corticosterone production by CAFs, but the phage-DNA-TLR9 signaling may exert other effects on CAFs and subsequently the tumor microenvironment, which requires further study; furthermore, the mechanism by which intratumor phages access intracellular TLR9 in CAFs remains to be determined. There are likely other translocating bacteria or polymicrobial interactions within tumors that also shape the microenvironment and affect antitumor immunity. While intratumor TLR9 targeting mitigated stress effects, off-target impacts and long-term safety in clinical contexts require further investigation. Future studies should validate these findings in diverse human cohorts and alternative models.

## RESOURCE AVAILABILITY

### Lead contact

Further information and requests for resources and reagents should be directed to and will be fulfilled by the lead contact, Melody Zeng (myz4001@med.cornell.edu).

### Materials availability

This study did not generate new unique reagents.

## STAR★METHODS

### EXPERIMENTAL MODEL AND STUDY PARTICIPANT DETAILS

#### Mice

Wild-type C57BL/6 (000664) mice were originally obtained from the Jackson Laboratory and subsequently maintained and expanded in-house by the Zeng laboratory under both germ-free (GF) and specific pathogen-free (SPF) conditions. Both male and female mice, aged 8 to 12 weeks, were included in each experiment. To generate B cell specific NR3C1 knock out mouse line, *Mb1/CD79a^Cre^* mice (020505) purchased from Jackson Laboratory were crossed with *NR3C1*^fl/fl^ (021021) mice provided by Mikala Egeblad (Johns Hopkins University). For orthotopic colorectal cancer model, *Cdx2*-Cre^ER^;*Apc*^fl/fL^ mice were kindly provided by Daniel Mucida (Rockefeller University). To generate a mouse line with an intestinal epithelial cell-specific deletion of *NR3C1*, *Villin*^Cre^ mice (004586) purchased from Jackson Laboratory were crossed with *NR3C1*^fl/fl^ mice (021021). IgG KO mouse line was generated by the Zeng lab. All *in vivo* experiments were performed independently at least twice. All animal experiments were conducted in accordance with protocols approved by the Institutional Animal Care and Use Committee (IACUC) at Weill Cornell Medicine (protocol number 2019-0004).

#### Mouse cancer cell lines

MC38 and B16F10 cells were cultured in complete DMEM media with 10% FBS and 1% Penicillin and streptomycin.

#### Human NAF and CAF cell lines

Paired normal (NAF) and cancer-associated fibroblast (CAF) cell lines derived from 3 human patients with lung or 3 patients with pancreatic ductal adenocarcinoma (PDAC), kindly provided by Dr. Moshe Oren (Weizmann Institute of Science, Israel), were cultured in DMEM F12 media.

#### Bacterial strains

Eg-gut (KZS13129), *L. johnsonii* were isolated from the intestine of a wild-type C57BL/6 mouse, and Eg-tumor (HBS23) was isolated from MC38 tumors from WT CUMS-induced mice. In addition, Kp-T8B and Kp-C8A *Klebsiella pneumoniae* bacterial strains were isolated from human colorectal tumors and adjacent normal colon tissues, respectively. All the three bacterial strains were cultured anaerobically in BHI broth at 37°C for 24 h.

#### Human samples

Human surgical resection samples from colon cancer patients were provided by the Cooperative Human Tissue Network (CHTN), which is funded by the National Cancer Institute. Other investigators may have received specimens from the same patients. Samples were received as entirely de-identified human specimens with diagnoses confirmed by medical records and trained pathologists. The protocol (20–07022342) was reviewed by the Weill Cornell Medicine Institutional Review Board and determined to meet the exemption category 4 of HHS 45 CFR 46.104(d). Additional oversight of the Cooperative Human Tissue Network is outlined at www.chtn.org. Samples included treatment-naive primary colon/rectal tumors and matched adjacent normal tissues from male (*n* = 10; age 52–86 years) and female (*n* = 6; age 59–86 years) patients undergoing surgical resection. Inclusion criteria included histologically confirmed colorectal adenocarcinoma with availability of matched adjacent non-malignant tissue mostly from sigmoidal region of colon, while patients with prior chemotherapy or radiotherapy treatment were excluded. Fresh tissues were shipped in MEM/DMEM media supplemented with penicillin/streptomycin, antifungal agents, and FCS and were processed immediately upon receipt. Tissue processing times following surgical resection ranged approximately from 12 to 18 h across specimens.

### METHOD DETAILS

#### Chronic unpredictable mild stress (CUMS) and tumor models

The mouse model of CUMS was adapted from previous studies.^4,98^ CUMS was induced in mice for 21 days with 2 randomly assigned stressors/day. The stressors included physical restraint in a 50 mL conical tube for 30 min, noise stress (80 decibeLs of white noise for 30 min), food deprivation (overnight), water deprivation (overnight), moist bedding (3–4 h), no bedding (3–4 h), removal of all bedding and addition of 30°C water (3–4 h), 30° cage tilt (12 h), and stroboscopic light (overnight). At day 7 of CUMS, blood was collected for measurement of plasma corticosterone by ELISA. For tumor model in CUMS mice, 5 × 10^5^ MC38 cells or 1 × 10^6^ melanoma B16F10 cells/mouse were injected in mice subcutaneously in 100μL PBS on the right flank at day 7 of CUMS.

Tumor size was measured two to three times weekly with calipers, blinded to group allocation when feasible using the formula, 1/2 (L*W^2^); L = Length and W= Width of tumor. Humane endpoints for euthanasia included any of the following criteria: tumor volume exceeding 2000 mm^3^, ulceration of the tumor, body weight loss greater than 20% from baseline, or signs of severe distress, moribund condition, or other signs of compromised health. Veterinary staff monitored the animals daily for these conditions. Mice were immediately sacrificed upon reaching any of these endpoints. No additional exclusion criteria were applied beyond these predefined humane endpoints. Mice were sacrificed 21 days post tumor inoculation. At day 21, 9 mice (Figures 1G and 3A, S8C) had tumor volumes exceeding 2000 mm^3^ and were euthanized immediately. At the previous measurement at day 18, all 9 tumors were between 1500 and 1800 mm^3^. The mice were monitored daily by veterinary staff and showed no clinical signs of distress, impaired mobility, or any other welfare concerns. They were not flagged by the veterinary team. The tumor volumes were next measured at day 21, at which point they exceeded 2000 mm^3^. These mice were euthanized immediately upon discovery. Notably, 5 of these 9 tumors belonged to mice in the chronic unpredictable mild stress + B cell depletion (CUMS+anti-CD20) group. Tumors in this cohort grew unusually rapidly between day 18 and day 21.

For the orthotopic colorectal cancer model, CUMS was induced in *Cdx2*-Cre^ER^ *Apc*^fl/fL^ mice at day minus 7 and thereafter, 0.8 mg/mouse tamoxifen (Cayman chemical) in corn oil was intraperitoneally injected at day 0 and day 3. After 4 weeks, mice were sacrificed to count and measure tumors.

#### B cell purification and *ex vivo* activation

Spleens were mashed and passed through 70-μm cell strainers to dissociate the cells into single-cell suspensions. Red blood cells (RBCs) were lysed with 2 mL of lysis buffer (150 mM NH4Cl, 10 mM KHCO3, 0.1 mM EDTA, pH 7.5) for 2 min at room temperature (RT). The lysis reaction was quenched by adding five volumes of B cell media (RPMI 1640 + l-glutamine (11875, GIBCO), 15% FBS (35-010-CV, Corning), and 1% penicillin–streptomycin (400-109, Gemini)). Following RBC lysis, B cells were isolated from total splenocytes by incubating with CD43 (Ly-48) MicroBeads (130-049-801, Miltenyi Biotec) in MACS buffer (500 mL PBS, 0.5% BSA, 0.5 M EDTA), followed by negative selection using LS Columns (130-042-401, Miltenyi Biotec). Naive B cells were seeded at 1 × 10^6^ per mL of B cell media and cultured *ex vivo* with combinations of LPS (50 ng/mL; L4130, Sigma-Aldrich), IL-4 (12.5 ng/mL; 404-ML-010, R&D Systems), and anti-mouse CD40 (1 μg/mL; 12-0401-82, eBioscience) in presence or absence of 2μM corticosterone (27840, Sigma-Aldrich). Cells were analyzed after 48 h of stimulation.

#### Mice immunization and tissue collection

Mice were immunized intraperitoneally with 100 μg of NP-KLH (SCBT) precipitated with Imject alum adjuvant (77161, Thermo Scientific), followed by a booster dose of 50 μg on day 7. Blood samples were collected via submandibular bleeding for the assessment of NP-specific plasma antibodies. On day 14, the spleen and mesenteric lymph nodes (MLN) were harvested for B cell analysis through flow cytometry.

#### ELISA for anti-tumor IgG

To measure anti-tumor plasma IgG, MC38 cells were heat killed by incubating at 56°C for 30 min in PBS and thereafter 50k cells were seeded in each well of 96-well ELISA plates (Nunc MaxiStop flat bottom 96-well, Thermo Scientific) overnight at 4°C. Next day, wells were blocked with blocking buffer (1xPBS, 1% BSA) for 2 h at RT. Plasma was diluted (1:10 ratio with blocking buffer) and 100 μL was added to each well at 4°C overnight. After washing, HRP conjugated anti-IgG Fc Fragment, 1:5000 dilution (A90-131P, Bethyl Laboratories) was added to plate. After a final wash, 100 μL 1-step Ultra TMB ELISA substrate (34028, Thermo Scientific) was added and the plate was incubated for 5 min before adding 50μL stop solution (SS04, Invitrogen). Absorbance was measured at 450 nm using a Spectramax iD3 plate reader.

#### ELISA for NP-specific plasma antibodies

For NP-specific antibody detection, ELISA plates were coated with 100 μL of 3 μg/mL NP_7_-BSA or NP_36_-BSA (*N*-5050L-10 and *N*-5050H-10, Biosearch Technologies) in 1× PBS (pH 8.0) and incubated overnight at 4°C. After washing and blocking with 1% BSA, 100 μL of plasma samples (diluted 1:100) were added per well and incubated overnight at 4°C. Following additional washes, HRP conjugated anti-IgG Fc Fragment was added and incubated for 2 h at RT. TMB substrate was then added, and the reaction was stopped with stop solution once the color developed. Absorbance was measured at 450 nm using a Spectramax iD3 plate reader.

#### Seahorse metabolic flux analysis

Naive B cells were isolated from spleens of designated groups by negative selection using CD43 beads. To prepare the cell culture plates (101085-004, Agilent) for the assay, Cell-Tak (354240, Corning) was diluted to ~23 μg/mL in 2 mL 0.1 M NaHCO_3_ at pH 8.0 and 30 μL of the solution is pipetted into each well. After 20 min, the solution was aspirated, and the wells were washed with 200 μL distilled water and subsequently air-dried for cell seeding. In parallel sensor cartridge was hydrated with 200 μL dH20 for minimum 4 h in a 37°C non-CO2 incubator overnight. After this, around 300k cells were resuspended in 180 μL of Seahorse minimal media and transferred into each well. Cells were spun at 300*g* for 1 min to allow them to stick to the wells. All the inhibitors provided in the kit were resuspended according to the manufacturer’s instructions (103020-100, Agilent). After about 30 min of incubation in a non-CO_2_ incubator, the plates were loaded into an XF96 Seahorse analyzer for ECAR and OCR measurements.

#### Corticosterone ELISA

Corticosterone level in blood plasma and homogenized tissue supernatant was done through competitive ELISA kit (RE52211, IBL international). Briefly, 20μL of sample and standard put into microtiter plate wells coated with anti-corticosterone antibody, followed by 200μL of enzyme conjugate, both components were mixed well, and incubation was done for 60 min. After an hour and 3× washing was given and 100 μL of substrate TMB solution was added with incubation of 15 min at RT. Later, the reaction was stopped by 50 μL of stop solution and absorbance (OD) was determined at 450nm with 620nm for background subtraction on Spectramax iD3 plate reader.

#### Elevated Plus Maze

Stress-associated behavior was assessed using the elevated plus maze (EPM). The apparatus consisted of two open arms (40 × 5 cm) and two closed arms (40 × 5 cm, enclosed by 30 cm-high walls), elevated 50 cm above the floor. Illumination at the center of the open arms was set to 100 lux. Each mouse was placed in the center area facing an open arm and allowed to freely explore the maze for 5 min. Arm entry was defined as all four paws crossing into an arm. Time spent in the open arms and closed arms was used to evaluate anxiety-like behavior, with decreased open arm time indicating increased stress level. The maze was cleaned with 70% ethanol and dried thoroughly between trials.

#### Intratumor bacterial culture

To assess intratumor microbial diversity, tumors were excised under sterile conditions on day 21. Tumor homogenates were then plated on De Man–Rogosa–Sharpe (MRS) and Brain Heart Infusion (BHI) agar plates under anaerobic conditions and incubated at 37°C for 24 h. Bacterial colonies were subsequently propagated in liquid media, and DNA was isolated from these cultures for PCR to amplify the 16s rRNA gene and Sanger sequencing for species identification.

#### Blood bacterial culture and CFU analysis

Peripheral blood was collected from mice by cheek bleed under sterile conditions in a Class II biosafety cabinet. Blood samples were centrifuged at 400 × g for 5 min to pellet immune and blood cells while retaining extracellular bacteria in the supernatant. The collected supernatant was diluted in BHI broth, serially diluted, and plated onto BHI agar plates under both aerobic and anaerobic conditions at 37°C for bacterial CFU quantification.

#### Eg and Lj conditioned media

Both bacteria were cultured anaerobically in BHI broth at 37°C for 24 h. To condition the media, the 24-h bacterial cultures were centrifuged at 5000g for 10 min, and the supernatant was filtered through a 0.2-micron filter. The filtered supernatant was then used for gavaging mice (200 μL/animal) and *in vitro* stimulation.

#### Bacterial and conditioned media gavage

Both Eg and Lj bacteria were cultured for 24 h in BHI, after which 10^8^ CFU/mL of each, suspended in 1xPBS, were orally gavaged into germ-free (GF) mice for monocolonization over a period of two weeks (200 μL/animal). Additionally, for conditioned media gavage, 200 μL of conditioned media was orally gavaged every other day throughout the experiment with BHI media as a control.

#### Intratumor injections

For intratumor conditioned media injections, Eg and Lj, conditioned media were prepared as already described. In parallel, MC38 tumor cells were injected subcutaneously at 5 × 10^5^ cells per mouse on day 0. Beginning on day 7, mice received intratumoral injections of Eg and Lj-conditioned media, or fresh BHI medium at 100 μL per mouse every 2 days until day 21. In addition, CUMS-exposed MC38 tumor-bearing mice received intratumoral injections of the TLR9 antagonist ODN 2088 at 10 μM as well 100ng Eg-DNA per mouse every 2 days beginning on day 7. Tumors and immune populations were analyzed at day 21.

#### Mitomycin C assay

To perform this assay, different Eg strains were grown overnight in BHI broth at 37°C. Next day, required bacterial culture was inoculated in 10mL of molten agarose (0.3–0.5%) with final bacterial CFU of 1 × 10^3^/mL and the whole inoculum was uniformly spread over fresh BHI agar plates to generate a lawn. Once the upper layer of agarose with bacteria solidified, different dilutions of Mitomycin C (sc-3514A, SCBT) starting from 2 μg/mL were spotted (5 μL/spot) over lawn colonies with BHI only as control and plates were incubated overnight at 37°C. The following day, the plates were examined for evidence of lytic phage activity in the form of a spot or clearing of the bacterial lawn.^99,100^

#### Organoid culture

Normal and CUMS mice intestinal tissues were cut into 2 mm segments, washed with cold DPBS, and subsequently resuspended in a gentle cell dissociation reagent (07174, StemCell Technologies). The tissues were then incubated under oscillation at room temperature. After incubation, the intestinal tissue was resuspended in cold DPBS containing 0.1% BSA and shaken using an up- and-down motion. The supernatants were collected, filtered through a 70 μm filter, and centrifuged at 4°C for 5 min at 250g. The crypt component was then seeded in Matrigel (356231, Corning), with the culture medium changed two to three times per week and subcultured after 7 to 10 days. Supernatants from these cultures were collected to determine corticosterone levels as a measure of the direct impact of CUMS on extra-adrenal corticosterone pathways in the gut. Additionally, organoids were cultured in the presence of corticosterone (2 μM) and bacterial supernatants to assess their direct effects.

#### *In vitro* FITC–Dextran permeability assay using colon organoids

Colon-derived organoids from control mice were cultured in organoid medium and treated with vehicle or corticosterone for 24 h. Organoids were then incubated with 4 kDa FITC–dextran for 1 h at 37°C. After incubation, organoids were washed and fresh medium was added before imaging. FITC–dextran uptake into the organoid lumen was assessed by fluorescence microscopy as a measure of epithelial permeability.

#### Antibiotic treatments

For antibiotic administration, mice were provided with drinking water containing a broad-spectrum antibiotic (Abx) cocktail (1 g/L ampicillin, 1 g/L neomycin sulfate, 1 g/L metronidazole, and 0.5 g/L vancomycin, all from Sigma-Aldrich), throughout the experiment to disrupt the gut microbiota. For intra-tumoral (i.t.) antibiotic administration, mice with tumors at around day 10 were injected daily with 100 μL of ampicillin (1 g/L in water) starting 10 days post tumor injection until the end of the experiment.

#### Acute and chronic DSS treatment model

Mice were subcutaneously injected with 5 × 10^5^ MC38 cells at day 0 and simultaneously administered either acute 2% DSS (160110, MP Biochemicals) for 7 days or chronic low-dose 0.2% DSS for 21 days *ad libitum* in drinking water. Gut permeability and circulating bacterial burden were assessed at days 7, 14, and 21, and immune populations were analyzed at the experimental endpoint on day 21.

#### Anti-PD-1 treatment in tumor-bearing mice

MC38 tumor-bearing mice were intraperitoneally administered 200 μg anti-PD-1 antibody per animal every 2 days starting from day 7 post tumor inoculation until sacrifice at day 21. Blood samples were collected at indicated time points, to assess circulating bacterial burden by CFU analysis. Tumor growth was monitored throughout the experiment, and mice were euthanized on day 21 for downstream analyses.

#### Immunofluorescence staining of mouse tumor tissues

Mouse tumor tissues were fixed overnight in 4% paraformaldehyde (Thermo Scientific). After fixation, tissues were washed with PBS, placed in 75% ethanol, and subsequently embedded in paraffin. Tissue sections of 5 μm thickness were mounted onto microscope slides. The sections were then permeabilized with 0.5% Triton X-100 for 15 min at room temperature. Antigen retrieval was performed using Citrate Buffer Antigen Retriever (Sigma-Aldrich) at 85°C for 30 min. After a washing step, the sections were blocked with 5% horse serum in PBS for 60 min at room temperature, followed by incubation with goat anti-mouse B220, CD3, and CD11c antibodies. After additional washing and processing steps, images were captured using a Zeiss AxioObserver inverted fluorescence microscope. The entire procedure was conducted at the Molecular Cytology Core Facility, Memorial Sloan Kettering Cancer Center.

#### 16*S* rRNA gene sequencing

Bacterial genomic DNA was extracted from fecal pellets using the E.Z.N.A. Stool DNA Kit (Omega Bio-tek), and from tumor homogenates using the DNeasy Blood and Tissue kit (Qiagen 69504). DNA amplicons of the V3-V4 region of the 16S ribosomal RNA (rRNA) gene were sequenced using an Illumina MiSeq instrument at Genomics Core Facility of the WCM Core Laboratories Center. The sequences were curated using mothur (v1.35), with sequences binned into OTUs at a >97% sequence similarity level. QIIME2 was used for demultiplexing, annotating the V3-V4 sequences, and removing potential contaminants.^82^ Amplicon sequence variants (ASVs) were generated using DADA2.^83^ LEfSe and Spearman ranking analyses were performed using mothur.^81,101^ The heatmap of ASV abundance was visualized using MeV.^84^

#### Eg whole genome sequencing (WGS)

WGS was performed by Plasmidsaurus (Eugene, Oregon). Genomic DNA was isolated from Eg-Tum and Eg-Gut as well as culture supernatants using the DNeasy Blood and Tissue Kit (Qiagen 69504). Amplification-free long-read sequencing libraries were prepared using Ligation Sequencing Kit V14 and thereafter sequenced using MinION R10.4.1 flow cell (Oxford Nanopore Technologies). The genomic and plasmid sequences for both tumor (HBS123) and gut strain (KZS13129) were annotated by prokka with the default parameters, followed by further fine annotation by Diamond search (blastp, e < 0.00001) with UniProt database uniref. 100. Phage sequences were detected by PhageScope. SNPs were detected by using snippy with fastq sequences of HBS123 and KZ13129, and the amino acid differences between these strains were determined by blastp. The plasmids were detected by blastn. These genomic sequences were also compared with publicly available reference strain sequences like fecal isolates EG3 and EG81 by using the RAST server. Proteins were binned into orthologue groups by roary (default) with E. faecalis 621 reference genomes and then resulted ortholog groups were further regrouped with ≥90% identity with Diamond. The domain structure of the proteins was determined by InterProScan. The paralog group numbers were based on the identity of domain structure predicted by InterProScan. Putative virulence proteins that are homologous to the reference set A of VFDB was determined by Diamond with e < 0.00001. Phylogenetic tree of HBS123 and KZS13129 with 230 complete and incomplete reference *E. gallinarum* genomic sequences, which were available on NCBI database (till Nov. 2024) was generated by iTol and using roary (default parameters).

#### B cell depletion

B cells were depleted by intraperitoneal administration of 200 μg/animal of anti-mouse CD20 mAb (clone 5D2, Genentech), with murine IgG2a antibody as an isotype control (GP120:9674, Genentech). The treatment began one week prior to tumor injection and was repeated twice per week throughout the duration of the experiment. B cell depletion was confirmed by assessing blood B cell abundance by flow cytometry.

#### FITC-dextran gut permeability assay

Food was removed from Control and CUMS mice for 4 h before being administered fluorescein isothiocyanate (FITC)–dextran, 500 mg/kg (FD4, Sigma-Aldrich) dissolved in PBS via oral gavage. After 1 h, blood was collected, and FITC intensity in the serum was measured using Spectramax iD3 plate reader (excitation at 485 nm, emission at 525 nm).

#### Mouse tumor tissue processing

Tumors were excised, chopped into smaller pieces, and resuspended in 7 mL of digestion media containing RPMI (21-870-076, Thermo Fisher) +10% FBS (10437028, Thermo Fisher), collagenase type 3, 0.8 mg/mL (LS004183, Worthington Biochemical), Dispase 0.6 mg/mL (17105041, Thermo Fisher) and DNase, 0.1mg/mL (DN5, Sigma-Aldrich) at 37°C for 30 min. To obtain single-cell suspension, the digested tumor was passed through a 70-micron nylon mesh using a syringe plunger in RPMI with 10% FBS. The single cell suspension was then centrifuged at 2000 rpm for 3 min. Cell pellets were washed using 20 mL of PBS. Subsequently, the cells were resuspended in 5 mL of 40% Percoll in RPMI with 10% FBS and centrifuged at 2000 rpm for 20 min without brake. Following this step, the cells were washed with 10 mL of PBS of and then resuspended in complete RPMI media and further used for flow cytometry and other assays.

#### Spleen and mesenteric lymph node (mLN) processing

Spleens and mesenteric lymph nodes (mLNs) were mechanically dissociated through a 70 μm cell strainer to generate single-cell suspensions. Splenocytes were centrifuged at 2,000 rpm for 4 min and resuspended in ACK lysis buffer (Quality Biological, 118-156-101) for 3 min at room temperature to remove red blood cells. Lysis was quenched with 25 mL PBS, followed by centrifugation at 2,000 rpm for 4 min. Splenocytes and mLN cells were subsequently washed twice with PBS before downstream analyses.

#### Human tissue processing and analysis

Surgical resection samples from the colon of patients with colon cancer, along with adjacent non-malignant colon tissue, were isolated by a trained pathologist. Single-cell suspensions from the non-malignant colon were prepared by incubating the tissue in stripping buffer containing 5% FBS, 1 mM EDTA (AM9261, Invitrogen), and 1 mM dithiothreitol (DTT) (R0861, Thermo Fisher) for 30 min at 37°C with shaking to remove the epithelial layer. The supernatants were then discarded. Both tumor and remaining adjacent tissues were mechanically dissociated using a sterile scalpel. The malignant tumor and non-adjacent tissue homogenate were then incubated for 1 h at 37°C with shaking in a digestion buffer containing 2 mg/mL collagenase D (COLLD-RO, Roche) and 0.1 mg/mL DNase I (DN25, Sigma-Aldrich) in RPMI with 5% FBS. The remaining tissues were filtered through a 70 μm cell strainer. The filtrate cells containing tumor single cells from malignant tissue and lamina propria cells from adjacent tissue, were enumerated and cryopreserved in 90% FBS and 10% DMSO (D8418, Sigma-Aldrich) for flow cytometry analysis later. Furthermore, some tissue from tumor and non-malignant colon were homogenized and centrifuged, after which the supernatant was used to estimate corticosterone level.

#### Flow cytometry

Cells from mouse and human tissues were isolated as described above. For intracellular cytokine staining, cells were stimulated *ex vivo* for 3–4 h with phorbol 12-myristate 13-acetate (PMA; 100 ng/mL; Sigma-Aldrich) and ionomycin (1 μg/mL; Sigma-Aldrich) in the presence of GolgiPlug (1:1000; BD Biosciences) at 37°C with 5% CO_2_. Following stimulation, cells were washed with cold PBS and incubated with Fc block for 15 min. Cells were then resuspended in FACS buffer (PBS containing 1% BSA) and stained with antibodies against surface markers and a viability dye for 20 min (all antibodies are listed in the Supplementary Key Resources Table). For intracellular staining, cells were subsequently fixed and permeabilized using the Foxp3/Transcription Factor Staining Buffer Set according to the manufacturer’s instructions (eBioscience). For surface-only staining, cells were incubated with Fc block followed by staining with surface antibodies in FACS buffer. After washing with FACS buffer, samples were acquired on a Cytek Aurora flow cytometer (Cytek) and analyzed using FlowJo software (Becton Dickinson). All antibody information is attached in Key Resource Table.

#### Human cancer-associated fibroblast culture with Eg-DNA stimulation

Paired normal (NAF) and cancer-associated fibroblast (CAF) cell lines derived from 3 human patients with lung or 3 patients with pancreatic ductal adenocarcinoma (PDAC), kindly provided by Dr. Moshe Oren (Weizmann Institute of Science, Israel), were cultured in DMEM F12 media and stimulated with Eg-Tum DNA (100ng/mL) in presence or absence of TLR9 antagonist, ODN 2088 (1μM), for 24 h. After 24 h, cells were lysed and 10μg lysate was used to determine corticosterone levels using competitive ELISA kit (RE52211, IBL international).

#### Intestinal organoid culture

Mice were sacrificed by CO_2_ asphyxiation, and the intestine was harvested from Control and CUMS SPF mice or germ-free mice monocolonized with Eg or Lj. The intestine was slit open longitudinally and chopped into ~2 mm segments, washed thoroughly with cold PBS, and subsequently resuspended in gentle cell dissociation reagent (07174, StemCell Technologies). The tissues were then incubated at 20 rpm for 15–20 min at room temperature. Thereafter, the supernatant was discarded, the intestinal tissue pieces were resuspended in cold PBS containing 0.1% BSA, pipetted up and down, and the supernatant was passed through a 70 μm cell strainer. This step was repeated 4–6 times to generate fractions with progressive enrichment of crypts. The collected fractions were centrifuged at 290 g at 4°C for 5 min. The pellet was washed twice in DMEM/F-12 containing 15 mM HEPES (36254, StemCell Technologies) by centrifuging at 200 g at 4°C for 3 min and 5 min, respectively. The pellet containing the crypts was then seeded in a 1:1 mixture of Matrigel (356231, Corning) and complete Intesticult Organoid Growth medium (mouse) (06000, StemCell Technologies) in a 24 well plate and incubated at 37°C with 5% CO_2_ in a humidified incubator. The organoid density was maintained such that each well of a 24 well plate contained ~150 organoids. The culture medium was changed 2–3 times per week and sub-cultured after 7 to 10 days. Additionally, organoids were cultured in the presence of corticosterone, 24 nM (27840, Sigma-Aldrich) for 24 h or bacterial culture supernatants with 1:10 dilution with organoid media for 72 h to assess their direct effects on the intestinal epithelium. Supernatants from these organoids were collected to determine corticosterone levels using competitive ELISA kit (RE52211, IBL international) as per manufacturer’s protocol, as a measure of the direct impact of CUMS or Eg/Lj monocolonization on extra-adrenal corticosterone synthesis by the intestinal epithelium. The organoids were harvested in TRI-zol reagent (15596026, Invitrogen) for RNA isolation and gene expression analysis.

#### Intestinal organoid stimulation

Intestinal organoids were cultured in Matrigel with complete IntestiCult organoid growth medium at 37°C with 5% CO_2_. After, 3–4 days of culture, organoids were stimulated with Eg-Tum DNA (50 ng/mL) in the presence or absence of the TLR9 antagonist ODN 2088 (1 μM) for 48 h. Organoid supernatants were then collected, to quantify corticosterone levels.

#### Preparation of bacterial culture supernatant for corticosterone estimation

Eg or Lj was cultured for 24 h (OD_600_ ~1.2) in BHI broth at 37°C under anaerobic conditions. The cells were centrifuged at 3000 rpm for 5 min and the supernatant was filtered through a 0.22 μm filter. Corticosterone levels in the bacterial culture supernatant was measured using ELISA following manufacturer’s protocol (RE52211, IBL international). Glucocorticoid receptor (GR) reporter assay was performed with the bacterial culture supernatant using a GR reporter assay kit following manufacturer’s protocol (21400, Cayman Chemical) to investigate the presence of bioactive glucocorticoids produced by the bacteria. Intestinal organoids were treated for 72 h with 1:10 dilution of the intact bacterial culture supernatant or the culture supernatant was pre-treated with 2 MBU of DNase0 (DB0715K, Biosearch Technologies), 1μg/mL proteinase K (19133, QIAGEN), or 0.001% Triton X-100 (T9284, Sigma-Aldrich).

#### RNA isolation and RT-qPCR

Intestinal organoids were harvested in TRIzol reagent and RNA was isolated following manufacturer’s protocol (Invitrogen). Isolated RNA (2 μg) was used for cDNA preparation using MultiScribe Reverse Transcriptase (4311235, Applied Biosystems). RT-qPCR was performed using PowerTrack SYBR Green qPCR master mix (A46012, ThermoFisher Scientific) on a CFX96 Touch real-time PCR detection system (Bio-Rad). The housekeeping gene glyceraldehyde 3-phosphate dehydrogenase (*Gapdh*) was used for normalization (primer information, see Table S1).

#### Explant culture from mouse intestine

Mice were sacrificed by CO_2_ asphyxiation and the intestine was harvested, washed by flushing with cold DPBS and slit open longitudinally. The intestine was incubated in RPMI containing penicillin-streptomycin for 1 h at RT. Thereafter, the intestine was cut into ~0.5 cm fragments and incubated in RPMI containing 10% FBS and penicillin-streptomycin (P4458, Sigma-Aldrich), 10 μL media per mg tissue for 24 h with the luminal surface facing upwards. Additionally, the media was supplemented with 1:10 dilution of intact bacterial culture supernatant or bacterial culture supernatant pretreated with DNase0, Proteinase K, or Triton X-100. After 48 h, the media was collected and centrifuged at 12,000 g for 5 min at 4°C. Corticosterone level in the media was estimated by ELISA following manufacturer’s protocol.

#### Preparation of tissue lysate for corticosterone ELISA

For mouse, ~1 cm of intestinal tissue and associated luminal contents were snap-frozen separately in liquid nitrogen. For human, associated muscles and fat were removed and a small piece of the tumor or normal adjacent tissue was snap-frozen in liquid nitrogen. The snap-frozen tissue pieces or luminal contents were homogenized by bead beating with metal beads in PBS (10 μL/mg). The lysate was centrifuged at 10,000 g for 5 min at 4°C. The supernatant was collected and used for estimating corticosterone levels by ELISA following manufacturer’s protocol.

#### Cloning of lentiviral vectors

Cloning of LentiGuide-Puro plasmids was performed following the LentiGuide-Puro cloning protocol.^102,103^ DNA oligos for non-targeting control single guide RNA (sgNT) and Tlr9-knockout single guide RNAs (Tlr9-KO sgRNA #1 and #2) were annealed and then ligated with the digested LentiGuide-Puro plasmid. The sgRNA sequences are listed in the Key Resources Table. Cloned LentiGuide-Puro plasmids were transformed into Stable Competent E. coli cells and cultured in LB broth containing 100 μg/mL ampicillin. The LentiGuide-Puro plasmids were extracted using the Midi-prep kit and then stored at −80°C for subsequent use. (Oligonucleotide information, see Table S1).

#### Production of lentivirus

Approximately 6×10^6^ HEK-293T cells were seeded in a 75T flask using DMEM supplemented with 10% FBS a day before transfection. Lentiviral transfer plasmid, psPAX2 packaging plasmid, and pMD2.G envelope plasmid were mixed in a total of 20 μg with a weight ratio of 4:3:1, respectively. According to the manufacturer’s instructions, the mixture was co-transfected into the seeded cells using Lipofectamine 3000. The media were replaced with fresh media 24 h after transfection. Lentivirus-containing media were harvested 48 h after media replacement, and cell debris was removed through centrifugation for 20 min at 3,000×g at 4°C. Lentivirus was concentrated through ultracentrifugation for 2 h at 100,000 × g at 4°C, and then stored at −80°C for subsequent use.

#### Generation of Tlr9-KO MC38 cells

Approximately 2 × 10^5^ MC38 cells were seeded in a 24-well plate using DMEM supplemented with 10% FBS and 1× Penicillin/streptomycin a day before transduction. Titrated lentivirus was mixed at a multiplicity of infection (MOI) of 10 with a final concentration of 10 μg/mL polybrene in the media. Two days after transduction, cells were selected using 10 μg/mL selection antibiotics for 3 days. MC38 cells stably expressing Cas9 and the sgRNA targeting Tlr9-KO were obtained by sequential lentiviral transduction and antibiotic selection. To confirm this, *Tlr9* target locus was PCR-amplified and subjected to Sanger sequencing. Indel formation resulting from non-homologous end joining (NHEJ) was quantified using sequence deconvolution tools (ICE and TIDE) to determine editing efficiency and confirm biallelic disruption of *Tlr9*. Biallelic Tlr9-KO polygenic MC38 cells were then cryopreserved at −196°C for subsequent use.

#### Single cell gene expression and VDJ recombination

MC38 tumor CD45^+^ cells from 8-week-old wild-type and CUMS mice were isolated by FACS sorting. Each sample was stained with anti-mouse CD45 and 1 μg of one of four hashtag antibodies: TotalSeq C0301 (M1/42, Biolegend, 155861), TotalSeq C0302 (M1/42, Biolegend, 155863), TotalSeq C0303 (M1/42, Biolegend, 155865), TotalSeq C0304 (M1/42, Biolegend, 155867). Staining was done in 200 μl for 30 min on ice then washed twice with FACS buffer. For 10× Genomics scRNA-seq, we generated three libraries that measure (1) mRNA transcript expression (RNA), (2) mouse-specific hashtag oligos (HTO), and (3) VDJ Recombination sequencing. A total of 22,000 cells per mouse were sorted and resuspended in PBS. Four individual samples were combined per well each using a different hashtag antibody. We proceeded with droplet generation, reverse transcription and library preparation per the protocols in the Chromium Single Cell 5′ v3 protocol (1000698, 10× Genomics) as per the manufacturer’s recommendations. After library prep, quality control was performed using a bioanalyzer (Agilent 2100 Bioanalyzer, Agilent Technologies). After passing quality control, the library was sequenced on the NovaSeqX Plus with 2 × 50-bp paired-end reads (Illumina) at a depth of 20,000 reads per cell for the gene expression data and 5,000 reads per sample for the HTO and VDJ libraries. Due to low amplification of the VDJ library for the tumor samples only secondary to few B cells in those samples, we adjusted to run 12 cycles in the sample index amplification PCR, rather than the suggested 8 cycles, with subsequent good yield.

#### Single-cell bioinformatics analyses

Raw sequencing data were processed using the CellRanger pipeline (version 9.0.0 10× Genomics) aligned to the mouse genome (mm10, provided by 10× Genomics, ver 3.0.0). Raw count data were loaded into R (v. 4.4.1) and analyzed with the Seurat R package (v 5.1.0). Wells were demultiplexed using the function HTODemux within the Seurat package with a cut-off HTO Margin of 1.5. The RNA dataset was then filtered to include only GEMs (gel beads in emulsion) with between 900 and 9,000 genes, log10 genes per UMI of 0.80, mitochondrial RNA <5% of total reads. Doublets (GEMs with two cells) were removed using the default settings of the DoubletFinder program (v2.0.4). We regressed out the contribution of the Cell Cycle by first running the CellCycleScoring function followed by the ScaleData function. In the end we recovered 62,206 singlets used for later analysis. Integration was performed using the IntegrateData function. The singlets expressed a median of 2219 genes expressed with a median of 7869 counts. Principal components analysis (PCA) was performed using the normalized SCT dataset (RunPCA function) and two-dimensional representations were generated using the top 50 PCA vectors RunUMAP function. Cells were clustered using the FindNeighbors function (top 50 PCA vectors) and FindClusters function. A final resolution of 0.4 was used to classify cells into gene expression clusters. The robustness of cluster classifications was validated using the significant DE lists.

We then used a publicly available murine scRNA seq dataset filtered to include spleen and thymus tissue from the TabulaMurisData program (V1.24) and the SingleR program (V3.20) to identify cell subtypes and filtered the data to only B cells. We verified that all the cells within the B cell subset expressed CD20 and had a productive VDJ recombination. We recovered a total of 4970 B cells used for later analysis. Results were plotted as Volcano plots using ggplot2 (V3.5.1) and as an input to perform GSEA (Gene set enrichment analysis) using the ClusterProfiler program (V3.2.0) and defining significantly differentially expressed genes with adjusted *p* value < 0.1 from DESeq2. We ran our DEG (differentially expressed gene) list against the M2 group (curated gene sets) from GSEA and reported the top significantly differentially expressed gene sets with biological relevance.

#### Online single-cell data processing

Single-cell RNA sequencing data were obtained from the GSE236384 dataset, which includes 67,759 cells derived from 17 colorectal cancer patients. The 2,000 most variable genes were selected for clustering analysis. Cells expressing fewer than 200 genes or more than 3,000 genes were excluded to ensure data quality. Data integration was performed using the single-cell variational inference (scVI) method. The top 3,000 variable genes were used for principal component analysis (PCA), and the top 30 principal components were applied to Uniform Manifold Approximation and Projection (UMAP) and clustering analyses. The FindAllMarkers function was used to identify cluster-specific marker genes. Visualization of the results, including violin plots, heatmaps, and dot plots, was generated using the VlnPlot, DoHeatmap, and DotPlot functions, respectively, with default parameters.

#### Reanalysis of brain tumor metagenomic data for phage enrichment

Publicly available metagenomic sequencing dataset (PRJNA1023304) on human glioma and metastatic brain tumors (*n* = 73; Group = Tumor) and oral samples (*n* = 151; Group = Control) were reanalyzed for detection of phage DNAs. To generate phage and control housekeeping protein queries, all phage proteins in the UniProt database were retrieved based on the taxonomic annotations in UniProt, and their domain structures were annotated using InterProScan with Pfam. Prophage proteins, showing homology to query sequences which have the domains that contains ‘phage’ in their description, were identified using DIAMOND blastp to determine their host species. Putative hosts were inferred from these results together with descriptions of experimentally identified hosts in the phage protein entries. Phage-specific proteins were further refined using the Pfam domain keyword ‘‘phage’’. Housekeeping proteins were extracted from bacterial proteins in the UniProt database using DIAMOND blastp, with core housekeeping proteins described in our previous study^104^ as queries. Identification of reads and calculation of the phage/genomic DNA ratio. R1 reads homologous to phage and control proteins were identified using DIAMOND blastx after removal of host (human) genomic sequences with Bowtie2. Reads were assigned to the most homologous query sequences and counted for the individuals which have more than 100000 phage reads (*n* = 10 or 11 for control or tumor group, respectively). Taxa were classified according to the most recent NCBI taxonomic classification and grouped at all taxonomic levels. The phage-to-control read ratio was calculated after matching the taxonomic bins of phage and control proteins. Statistical significance was calculated in R and reported as Signal-to-noise ratio (SNR) and *p*- values.

### QUANTIFICATION AND STATISTICAL ANALYSIS

Statistical analyses were conducted using GraphPad Prism 10. Differences between two groups were evaluated using unpaired t test (parametric), unpaired t test with Welch’s correction, or Mann-Whitney U test (nonparametric). Differences between three or more groups were evaluated using one or two-way analysis of variance (ANOVA) (parametric) or Kruskal-Wallis test (non-parametric) with multiple comparisons tests (Tukey’s, Dunn’s, Dunnett’s T3, and Holm-Šidák’s) for adjusted P values. Spearman’s rank correlation coefficient was used for correlation of variables between groups. Differences with p values < 0.05 were considered significant.

## Supplementary Material

MMC1

Supplementary data related to this article can be found online at https://doi.org/10.1016/j.ccell.2026.06.004.

## Figures and Tables

**Table:** KEY RESOURCES TABLE

REAGENT or RESOURCE	SOURCE	IDENTIFIER
Antibodies		
Alexa Fluor-700 anti-mouse CD45, clone 30-F11	BioLegend	Cat# 103128; RRID: AB_493715
Alexa Fluor-488 anti-mouse NR3C1, clone BuGR2	Thermo Fisher	Cat# 53-6189-82; RRID: AB_2811875
Pacific Blue anti-mouse B220, clone RA3-6B2	BioLegend	Cat# 103227; RRID: AB_492876
PerCP-Cy5.5 anti-mouse/mouse GL-7, clone GL7	Biolegend	Cat# 144610; RRID: AB_2562979
BUV593 anti-mouse CD95 (FAS), clone Jo2	BD Biosciences	Cat# 741292; RRID: AB_2870823
BUV395 anti-mouse IgD, clone 11-26c.2a	BD Biosciences	Cat# 564274; RRID: AB_2738723
BUV805 anti-mouse CD38, clone 90	BD Biosciences	Cat# 741955; RRID: AB_2871263
BV785 anti-mouse CD69, clone H1.2F3	BioLegend	Cat# 104543; RRID: AB_2629640
Alexa Fluor 700 anti-mouseCD21/CD35, clone 7.00E+09	BioLegend	Cat# 123431; RRID: AB_2860649
PE/Cy7 anti-mouse CD23, clone B3B4	BioLegend	Cat# 101614; RRID: AB_2103036
BV650 anti-mouse CD138, clone 281-2	BioLegend	Cat# 142517; RRID: AB_2562404
Alexa Fluor 647 CXCR4, clone L276F12	BioLegend	Cat# 146503; RRID: AB_2562590
FITC anti-mouse Granzyme B, clone QA18A28	BioLegend	Cat# 396404; RRID: AB_2801073
APC anti-mouse Perforin, clone S16009B	BioLegend	Cat# 154404; RRID: AB_2721465
PE anti-mouse PD-1, clone RMP1-30	BioLegend	Cat# 109103; RRID: AB_313420
PE anti-mouse IgA, clone mA-6E1	eBioscience	Cat# 12-4204-82; RRID: AB_465917
PerCP-Cy5.5 anti-mouse CXCR5, clone L138D7	BioLegend	Cat# 145508; RRID: AB_2561972
FITC anti-mouse CD127, clone A7R34	Invitrogen	Cat# 11-1271-81; RRID: AB_465194
PE Anti-NP(4-Hydroxy-3-nitrophenylacetyl	Biosearch Technologies	Cat# N-5070-1
BV510 anti-mouse IgM, clone RMM-1	BioLegend	Cat# 406531; RRID: AB_2650758
FITC anti-mouse IgG, clone	eBioscience	Cat# 11-4011-85; RRID: AB_465218
PerCP-Cy5.5 anti-mouse CD25, clone PC61	BioLegend	Cat# 102029; RRID: AB_893291
Brilliant Violet 711 anti-mouse CD3e, clone 145-2C11	BioLegend	Cat# 100349; RRID: AB_2565841
Brilliant Violet 510 anti-mouse CD4, clone RM4-4	BioLegend	Cat# 116025; RRID: AB_2800580
PerCP-eFluor 710 anti-mouse CD8a, clone 53-6.7	Invitrogen	Cat# 46-0081-80; RRID: AB_1834434
BV 605 anti-human CD25, clone CD25-4E3	eBioscience	Cat# 406-0257-42; RRID: AB_3099375
e450 anti-mouse gdTCR, clone eBioGL3	eBioscience	Cat# 48-5711-80; RRID: AB_2574070
BV421 anti-mouse F4/80, clone BM8	BioLegend	Cat# 123131; RRID: AB_10901171
BV650 anti-mouse NK1.1, clone PK136	BioLegend	Cat# 108735; RRID: AB_11147949
PE-Cyanine5.5 anti-mouse/rat FOXP3, clone FJK-16s	Invitrogen	Cat# 35-5773-82; RRID: AB_11218094
PE-CF594 anti-mouse RORgT, clone Q31-378	BD Biosciences	Cat# 562684; RRID: AB_2651150
BV421 anti-mouse IL-17A, clone TC11-18H10.1	BioLegend	Cat# 506926; RRID: AB_2632611
PE anti-mouse CD206, clone MR6F3	Invitrogen	Cat# 12-2061-82; RRID: AB_2637422
APC anti-mouse CD11b, clone M1/70	BioLegend	Cat# 101212; RRID: AB_312795
PE-Cy5 anti-mouse CD11c, clone N418	eBioscience	Cat# 15-0114-82; RRID: AB_468717
PE-Cyanine7 anti-mouse Ly-6G (Gr1), clone RB6-8C5	eBioscience	Cat# 25-5931-82; RRID: AB_469663
Brilliant Violet 650 anti-mouse IFN-γ, clone XMG1.2	BioLegend	Cat# 505832; RRID: AB_2734492
PE/Cyanine7 anti-mouse TNF-α, clone MP6-XT22	BioLegend	Cat# 506323; RRID: AB_2204356
BV711 anti-human FAS, clone DX2	BioLegend	Cat# 305643; RRID: AB_2629740
Alexa Fluor 700 anti-human CD138, clone MI15	BioLegend	Cat# 356511; RRID: AB_2562638
PerCP anti-human CD27, clone M-T271	BioLegend	Cat# 356431; RRID: AB_2650752
PE/Cy7 anti-human IgD, clone IA6-2	BioLegend	Cat# 348209; RRID: AB_10683460
PE anti-human CD38, clone HB7	BioLegend	Cat# 356603; RRID: AB_2561899
Pacific Blue anti-mouse/human CD11b, clone M1/70	BioLegend	Cat# 101224; RRID: AB_755986
PE anti-human CD14, clone M5E2	BioLegend	Cat# 301850; RRID: AB_2564138
PE/Cy7 anti-human CD11c, clone BU15	Invitrogen	Cat# MA5-38749; RRID: AB_2898661
Alexa Fluor anti-human CD4, clone OKT4 (OKT-4)	Invitrogen	Cat# 56-0048-82; RRID: AB_657741
BV711 anti-human CD3, clone UCHT1	Invitrogen	Cat# 407-0038-42; RRID: AB_2921023
BV805 anti-human HLA-DR, clone LN3	Invitrogen	Cat# 368-9956-42; RRID: AB_2896177
Alexa Fluor 647 anti-human CD68, clone eBioY1/82A	Invitrogen	Cat# 51-0689-42; RRID: AB_2848399
APC anti-human CD45, clone HI30	BioLegend	Cat# 304011; RRID: AB_314399
Pacific Blue anti-human CD19, clone SJ25C1	BioLegend	Cat# 363035; RRID: AB_2632786
Anti-mouse CD16/32 Fc block	Biolegend	Cat# 101302; RRID: AB_312801
Anti mouse CD40, clone 1C10	eBioscience	Cat# 12-0401-82; RRID: AB_465649
Anti-mouse IgG-HRP, polyclonal	Bethyl Laboratories	Cat# A90-131P; RRID: AB_67175
Murine IgG2a antibody, clone GP120:9674	Genentech	Cat# GP120:9674
Anti-mouse CD20 mAb, clone 5D2	Genentech	Cat# 5D2
Anti mouse PD-1, clone RMP1-14	BioXCell	Cat# BE0146; RRID: AB_10949053
TotalSeq^™^-C0301 anti-mouse Hashtag 1, clone M1/42	BioLegend	Cat# 155861; RRID: AB_2800693
TotalSeq^™^-C0302 anti-mouse Hashtag 2, clone M1/42	BioLegend	Cat# 155863; RRID: AB_2800694
TotalSeq^™^-C0303 anti-mouse Hashtag 3, clone M1/42	BioLegend	Cat# 155865; RRID: AB_2800695
TotalSeq^™^-C0304 anti-mouse Hashtag 4, clone M1/42	BioLegend	Cat# 155867; RRID: AB_2800696
Bacterial and virus strains		
*Enterococcus gallinarum* tumor isolate (Eg-Tum)	This study	–
*Enterococcus gallinarum* gut isolate (Eg-Gut)	This study	–
*Lactobacillus johnsonii*	Katherine Z. Sanidad et al.^79^	–
5-alpha Competent *E. coli*	New England Biolabs	C2987H
Stable Competent *E. coli*	New England Biolabs	C3040H
*Klebsiella pneumoniae* (C8A-Human CRC normal colon derived)	This study	–
*Klebsiella pneumoniae* (T8B-Human CRC tumor derived)	This study	–
Biological samples		
Human colon cancer tissue samples (and adjacent non-malignant tissue)	National Cancer Institute-supported Cooperative Human Tissue Network (NCI-CHTN) Program	–
Chemicals, peptides, and recombinant proteins		
Fluorescein isothiocyanate (FITC)–dextran	Sigma-Aldrich	FD4
E780 viability dye	Invitrogen	65-0865-14
Bovine Serum Albumin (BSA)	Sigma-Aldrich	A2153
NP_7_-BSA	Biosearch Technologies	N-5050L-10
NP_36_-BSA	Biosearch Technologies	N-5050H-10
1-step Ultra TMB ELISA substrate	Thermo Scientific	34028
ELISA stop solution	IBL INTERNATIONAL	RE52211_70
Collagenase D	Roche	COLLD-RO
Collagenase type 3	Worthington Biochemical	LS004183
Phorbol 12-myristate 13-acetate	Sigma Aldrich	P8139
Ionomycin	Sigma Aldrich	I0634
GolgiPlug Protein Transport Inhibitor	BD Biosciences	51-2301KZ
Dispase	Thermo Fisher	17105041
Proteinase K	Qiagen	19133
MultiScribe Reverse Transcriptase	Applied Biosystems	4311235
DNase0	Biosearch technologies	DB0715K
DNase I	Sigma-Aldrich	DN25
Percoll	Cytiva	17089101
Lipopolysaccharides (LPS)	Sigma-Aldrich	L4130
IL-4	R&D Systems	404-ML-010
Mitomycin C	SCBT	sc-3514A
Corticosterone	Sigma-Aldrich	27840
NP-KLH (4-Hydroxy-3-nitrophenylacetyl-Keyhole Limpet Hemocyanin)	Santa Cruz Biotechnology	sc-396478
Ethylenediaminetetraacetic acid (EDTA)	Thermo Fisher	15575020
Ampicillin	Sigma-Aldrich	A0166
Neomycin sulphate	Sigma-Aldrich	4801
Metronidazole	Sigma-Aldrich	M3761
Vancomycin	Sigma-Aldrich	J62790.06
Paraformaldehyde (PFA)	Thermo Scientific	A11313.22
Triton X-100	Sigma-Aldrich	T9284
Dithiothreitol (DTT)	Sigma-Aldrich	R0861
Dimethyl sulphoxide (DMSO)	Sigma-Aldrich	D8418
ODN 2088 (TLR9 antagonist)	InvivoGen	tlrl-2088
ODN 1585 (TLR9 agonist)	InvivoGen	tlrl-1585
Ultrapure Agarose	invitrogen	16500-500
Ethanol	Fischer bioreagents	64-17-5
BsmBI-v2	New England Biolabs	R0739S
Quick CIP	New England Biolabs	M0525S
T4 Polynucleotide Kinase	New England Biolabs	M0201S
T4 DNA Ligase	New England Biolabs	M0202S
Lipofectamine 3000	Invitrogen	L3000008
Polybrene	Sigma-Aldrich	107689
Blasticidin	Gibco	A1113903
Puromycin	Gibco	A1113803
Dextran Sulfate Sodium Salt, colitis grade	MP Biochemical	160110
Critical commercial assays		
Corticosterone competitive ELISA kit	IBL international	RE52211
Corning Cell-Tak Solution	Corning	354240
Foxp3/Transcription Factor Staining Buffer Set	eBioscience	00-5523-00
E.Z.N.A. Stool DNA Kit	Omega Bio-tek	D4015-02
DNeasy Blood and Tissue Kit	Qiagen	69504
Ligation Sequencing Kit V14	Oxford Nanopore Technologies	SQK-LSK114
MinION R10.4.1 flow cell	Oxford Nanopore Technologies	FLO-MIN114
GR reporter assay kit	Cayman Chemical	21400
MultiScribe Reverse Transcriptase	Applied Biosystems	4311235
QIAquick Plasmid Midi Kit	Qiagen	12145
QIAquick Gel Extraction Kit	Qiagen	28706
PowerTrack SYBR Green qPCR master mix	ThermoFisher Scientific	A46012
Chromium GEM-X Single Cell 5 Feature Barcode Kit v3	10x Genomics	1000703
Chromium GEM-X Single Cell 5’ Kit v3	10x Genomics	1000695
Chromium GEM-X Single Cell 5’ Chip Kit v3	10x Genomics	1000698
Dual Index Kit TN Set A	10x Genomics	1000250
Chromium Single Cell Mouse BCR Amplification Kit	10x Genomics	1000255
Dual Index Kit TT Set A	10x Genomics	1000215
Library Construction Kit C	10x Genomics	1000694
Deposited data		
Intratumor B cell scRNAseq dataset	This study	GSE327985
Intratumor B cell BCRseq dataset	This study	GSE327985
16S analysis of control and CUMS mice	This study	PRJNA1451867
UniProt database uniref100	Steinegger M et al.^80^	https://www.uniprot.org/help/uniref
Mouse genome mm10 (v 3.0.0)	10X Genomics	https://www.ncbi.nlm.nih.gov/datasets/genome/GCF_000001635.20/
Human colorectal cancer scRNAseq dataset	Stary, V., et al.^40^	GSE236384
Human brain tumor and metastasis metagenomic dataset	Morad, G., et al.^59^	PRJNA1023304
Experimental models: Cell lines		
MC38 cells	Sigma-Aldrich	SCC172
B16F10 cells	ATCC	CRL-6475
Normal and cancer-associated human cell lines	Lung and pancreatic cancer patients	NAF/CAF101
Human: HEK-293T	ATCC	CRL-3216
Experimental models: Organisms/strains		
Wildtype C57BL/6 mice	Jackson Laboratory	000664
Mb1^Cre^ C57BL/6 mice	Jackson Laboratory	020505
*Nr3c1^flox/flox^* mice	Jackson Laboratory	021021
*Villin*^Cre^ C57BL/6 mice	Jackson Laboratory	004586
*Cdx2*-Cre^ER^;*Apc*^fl/fl^ C57BL/6 mice	Daniel Mucida laboratory	–
IgG KO C57BL/6 mice	Melody Y. Zeng laboratory	–
Recombinant DNA		
pMD2.G	Addgene	12259
psPAX2	Addgene	12260
LentiCas9-Blast	Addgene	52962
LentiGuide-Puro	Addgene	52963
Software and algorithms		
Mothur v1.35	Schloss PD et al.^81^	https://mothur.org/wiki/mothur_v.1.35.0/
QIIME2	Bolyen E et al.^82^	https://qiime2.org/
DADA2	Callahan BJ et al.^83^	https://benjjneb.github.io/dada2/
MeV	Saeed AI et al.^84^	https://sourceforge.net/projects/mev-tm4/
Prokka	Seemann T et al.^85^	https://github.com/tseemann/prokka
Diamond	Buchfink B **et al.**^86^	https://bio.tools/diamond
Blastp	NCBI	https://blast.ncbi.nlm.nih.gov/
Blastn	NCBI	https://blast.ncbi.nlm.nih.gov/Blast.cgi
PhageScope	Wang RH et al.^87^	https://phagescope.deepomics.org/
Snippy	NIH HPC Systems	https://hpc.nih.gov/apps/snippy.html
RAST	Aziz RK et al.^88^	https://rast.nmpdr.org/
Roary	Page AJ **et al.**^89^	https://hpc.nih.gov/apps/roary.html
InterProScan	Jones P et al.^90^	https://www.ebi.ac.uk/interpro/search/sequence/
iTol	Letunic I et al.^91^	https://itol.embl.de/
CellRanger v9.0.0	Zheng GX et al.^92^	https://github.com/10XGenomics/cellranger
R v4.4.1	R project	https://www.r-project.org/
Seurat v5.1.0	Hao Y et al.^93^	https://cran.r-project.org/web/packages/Seurat/index.html
DoubletFinder v2.0.4	McGinnis CS et al.^94^	https://rpubs.com/eileenowens/doubletfinder
TabulaMurisData v1.24	Bioconductor	https://bioconductor.org/packages/release/data/experiment/html/TabulaMurisData.html
SingleR v3.20	Aran D et al.^95^	https://github.com/SingleR-inc/SingleR
ggplot2 v3.5.1	R Project	https://cran.r-project.org/web/packages/ggplot2/index.html
ClusterProfiler v3.2.0	Xu S et al.^96^	https://bioconductor.org/packages/release/bioc/html/clusterProfiler.html
DESeq2	Love MI et al.^97^	https://github.com/thelovelab/DESeq2
Other		
RPMI 1640	Sigma Aldrich	R0883
ACK lysis buffer	Quality Biological	118-156-101
DMEM	Gibco	11965-092
DMEM/F12	Invitrogen	11320033
L-glutamine	Gibco	11875
Fetal Bovine Serum (FBS)	Gibco	10437-028
De Man–Rogosa–Sharpe (MRS) broth	Sigma Aldrich	69966
Brain Heart Infusion (BHI) broth	Teknova	101324
Luria-Bertani (LB) Broth Miller	BD Difco	244620
Opti-MEM	Gibco	31985062
Gentle cell dissociation reagent	StemCell Technologies	07174
Matrigel	Corning	356231
Imject alum adjuvant	Thermo Scientific	77161
Penicillin-streptomycin	Sigma-Aldrich	P4458
2-[4-(2-hydroxyethyl)piperazin-1-yl]ethanesulfonic acid (HEPES)	StemCell Technologies	36254
Complete Intesticult Organoid Growth medium (mouse)	StemCell Technologies	06000
TRIzol reagent	Invitrogen	15596026
Phosphate Buffered Saline (PBS)	Thermo Fisher	20012043
Dulbecco’s Phosphate Buffered Saline (DPBS)	Gibco	11965-092
CD43 (Ly-48) Microbeads	Miltenyi Biotec	130-049-801
LS columns	Miltenyi Biotec	130-042-401
Seahorse XFp Cell Mito Stress Test Kit	Agilent	103010-100
Seahorse XFp FluxPak	Agilent	103022-100
Seahorse XF RPMI medium	Agilent	103576-100
Seahorse XF 1M glucose solution	Agilent	103577-100
Seahorse XF 100mM pyruvate solution	Agilent	103578-100
Seahorse XF 200mM glutamine solution	Agilent	103579-100

## Data Availability

This study used both original and previously published sequencing and metagenomic data. Datasets original to this study include the following: 16S rRNA sequencing data of intratumor and fecal microbiome of Control or CUMS mice inoculated with MC38 tumor cells, available on SRA with accession number PRJNA1451867; whole-genome sequencing of E. gallinarum and K. pneumoniae isolates, available on SRA with accession number PRJNA1451867; and single-cell RNA-seq data of intratumor B cells, available on GEO with accession number GSE327985 (this includes both gene expression and VDJ datasets). Previously published datasets include the following: Single-cell RNA-seq data of human colorectal tumors and adjacent healthy tissue, from a study by Stary et al.,^40^ available on GEO with accession number GSE236384; and metagenomic analysis of glioma and metastatic brain tumors, from a study by Morad et al.,^59^ available on BioProject with accession number PRJNA1023304. This study did not generate original code. Any additional information required to reanalyze the data reported in this paper is available from the lead contact upon request.

## Highlights

- Chronic stress leads to the translocation of gut *Enterococcus gallinarum* to tumors
- Eg phage DNA induces glucocorticoid production by cancer-associated fibroblasts via TLR9
- Intratumor glucocorticoids suppress anti-tumor GC B cells via the glucocorticoid receptor
- Targeting intratumor TLR9 or Eg rescues GC B cells and restores anti-tumor immunity
