# Supplementary material for: Chronic stress unleashes an intratumor phage-fibroblast-B cell circuit to promote tumor growth
Source: Cancer Cell. Author manuscript; Available in PMC 2026 Aug 17. (PMC13479879; doi:10.1016/j.ccell.2026.06.004)
Supplement: Supplementary file 1 [file NIHMS2191533-supplement-MMC1.pdf]

Cancer Cell, Volume *44*

Supplemental information

Chronic stress unleashes an intratumor

phage-fibroblast-B cell circuit

to promote tumor growth

Hilal Bashir, Katherine Z. Sanidad, Purnima Ravisankar, Kelly M. Banks, Avipsa Bose, Shui Yu, Joshua J.C. McGrath, Hannah C. Carrow, Taeyul K. Kim, Chloe A. Troxell, Julia A. Brown, Lucy R. Hart, Jeremy Goc, Mikala Egeblad, Jayanta Chaudhuri, Han Sang Kim, Patrick C. Wilson, David C. Lyden, Irina R. Matei, Naohiro Inohara, Gregory F. Sonnenberg, and Melody Y. Zeng

Figure S1

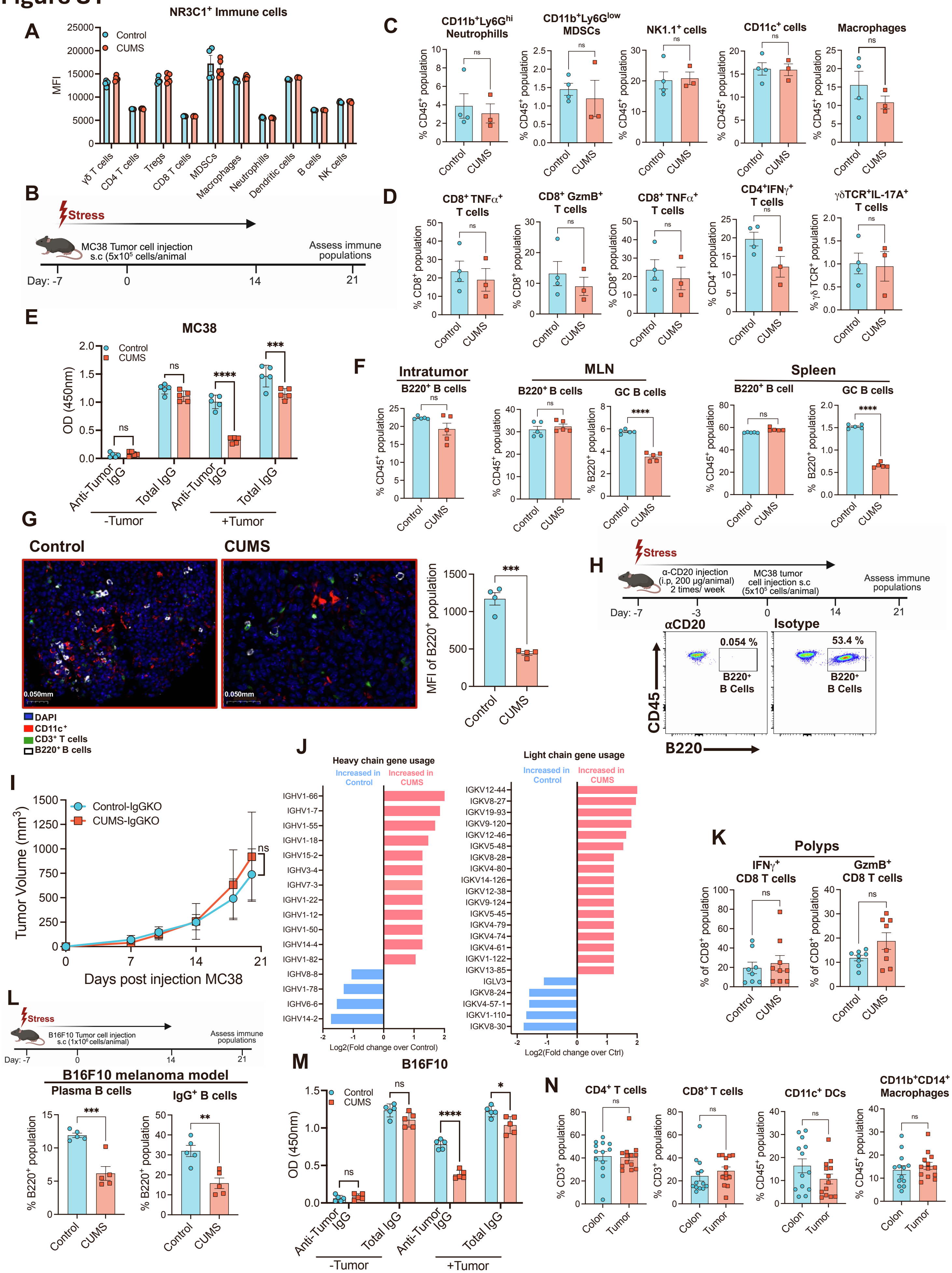

A
NR3C1+ Immune cells
Control
CUMS
MFI
γδ T cells
CD4 T cells
Tregs
CD8 T cells
MDSCs
Macrophages
Neutrophills
Dendritic cells
B cells
NK cells
B
Stress
MC38 Tumor cell injection
s.c (5x10⁵ cells/animal)
Assess immune
populations
Day: -7
0
14
21
C
CD11b+Ly6Ghi
Neutrophills
CD11b+Ly6Glow
MDSCs
NK1.1+ cells
CD11c+ cells
Macrophages
% CD45+ population
ns
Control
CUMS
D
CD8+ TNFα+
T cells
CD8+ GzmB+
T cells
CD4+IFNγ+
T cells
γδTCR+IL-17A+
T cells
% CD8+ population
% CD4+ population
% γδ TCR+ population
E
MC38
OD (450nm)
Anti-Tumor
IgG
Total IgG
-Tumor
+Tumor
ns
****
***
F
Intratumor
B220+ B cells
MLN
GC B cells
Spleen
B220+ B cell
% B220+ population
****
G
Control
CUMS
0.050mm
DAPI
CD11c+
CD3+ T cells
B220+ B cells
MFI of B220+ population
***
H
Stress
α-CD20 injection
(i.p, 200 μg/animal)
2 times/ week
MC38 tumor
cell injection s.c
(5x10⁵ cells/animal)
Assess immune
populations
Day: -7
-3
0
14
21
αCD20
Isotype
0.054 %
53.4 %
B220+
B Cells
CD45
B220
I
Control-IgGKO
CUMS-IgGKO
Tumor Volume (mm3)
Days post injection MC38
ns
J
Heavy chain gene usage
Increased in
Control
Increased in
CUMS
IGHV1-66
IGHV1-7
IGHV1-55
IGHV1-18
IGHV15-2
IGHV3-4
IGHV7-3
IGHV1-22
IGHV1-12
IGHV1-50
IGHV14-4
IGHV1-82
IGHV8-8
IGHV1-78
IGHV6-6
IGHV14-2
Log2(Fold change over Control)
Light chain gene usage
IGKV12-44
IGKV8-27
IGKV19-93
IGKV9-120
IGKV12-46
IGKV5-48
IGKV8-28
IGKV4-80
IGKV14-126
IGKV12-38
IGKV9-124
IGKV5-45
IGKV4-79
IGKV4-74
IGKV4-61
IGKV1-122
IGKV13-85
IGLV3
IGKV8-24
IGKV4-57-1
IGKV1-110
IGKV8-30
Log2(Fold change over Ctrl)
K
Polyps
IFNγ+
CD8 T cells
GzmB+
CD8 T cells
% of CD8+ population
L
Stress
B16F10 Tumor cell injection
s.c (1x10⁶ cells/animal)
Assess immune
populations
B16F10 melanoma model
Plasma B cells
IgG+ B cells
% B220+ population
***
**
M
B16F10
OD (450nm)
*
N
CD4+ T cells
CD8+ T cells
CD11c+ DCs
CD11b+CD14+
Macrophages
% CD3+ population
% CD45+ population
Colon
Tumor

Figure S1. Chronic stress accelerates tumor progression by dampening anti-tumor B cell responses (related to Figure 1)

(**A**) Mean fluorescent intensity of Glucocorticoid Receptor (NR3C1) in different immune populations in spleen at day 21 of CUMS (n=5 mice/group).
(**B**) Schematic of the CUMS+MC38 colorectal cancer model.
(**C-D**) Population of different innate (**C**) and T cell (**D**) populations at intratumor sites (n=3-4 mice/group).
(**E**) Plasma anti-tumor as well as total IgG in MC38 tumor bearing mice (n=5 mice/group).
(**F**) Population of total B cells and GC B cells in the MLN, spleen and tumors (n=5 mice/group).
(**G**) Immunohistochemistry staining of day 21 tumors, showing intratumor $B220^+$ B cells, $CD11c^+$ dendritic cells and $CD3^+$ T cells with bar plot for mean fluorescence intensity of the $B220^+$ intratumor B cell population (n=4 mice/group).
(**H**) Experimental layout for B cell depletion in CUMS or Control mice with MC38 cell inoculation and validation of depletion of peripheral blood B cells after 3 days of anti-CD20 (200μg/animal).
(**I**) MC38 tumor growth kinetics in IgG-deficient (IgG KO) Control and CUMS mice (n=4 mice/group).
(**J**) Bar plots showing differential heavy and light chain gene usage in Control vs CUMS intratumor B cells (n=2 mice/group).
(**K**) Polyp $IFN\gamma^+$ and $granzyme^+$ CD8 T cells (n=8-9 mice/group).
(**L**) Abundance of intratumor $B220^+CD38^+IgD^-$ plasma cells, and $B220^+IgG^+$ B cells in melanoma tumors (n=5 mice/group).
(**M**) Plasma anti-tumor as well as total IgG in B16F10 tumor bearing mice (n=5 mice/group).
(**N**) abundance of different innate and adaptive immune cell populations in human colon tumors in comparison to adjacent normal colon tissue (n=13 patients/group).
Graphs show mean ± SEM . For A-G and K, each point represents one mouse, while for N each point represents one patient patient. C, D, F, G, K, L, N: two-tailed unpaired Student’s t test; A, E, I, M: two-way ANOVA with Šídák's multiple comparisons: ns=non-significant, *$p<0.05$, **$p<0.01$, ***$p<0.005$ and ****$p<0.0001$.

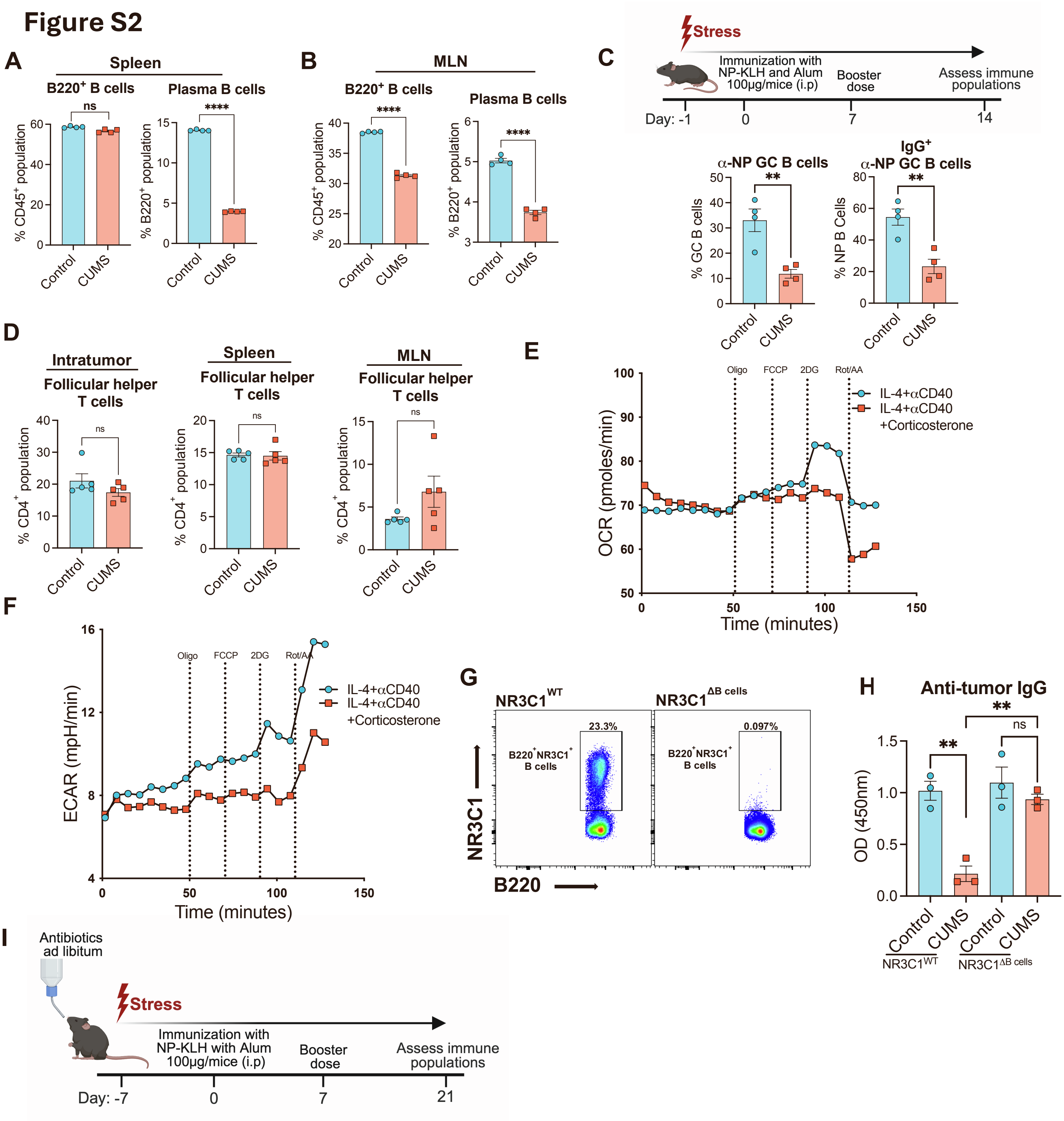

Figure S2. CUMS impairs antigen-specific B cell response in a gut microbiota-dependent manner (related to Figure 2)

(**A-B**) Abundance of $CD45^+B220^+$ B cells and B220+CD38+IgD- plasma cells in the spleen (**A**) and MLN of CUMS or Control mice (**B**) after 21 days of CUMS (n=4 mice/group).

(**C**) Experimental layout of intraperitoneal injection of the B cell immunogen NP-KLH (100 µg/animal) in Control or CUMS mice, percentage population of NP specific and IgG^+ NP specific GC B cells (n=4 mice/group).

(**D**) Abundance of Tfh ($CD3^+CD4^+BCL6^+CXCR5^+$) cells in MC38 tumor (left), and spleen (middle) as well as MLN (right) of CUMS or Control mice after 21 days of CUMS (n=5 mice/group).

(**E**) Representative trace of oxygen consumption rate (OCR) of three independent experiments after stimulation of naive B cells isolated from Control or day-21 CUMS mice in the presence or absence of IL-4 and CD40 (n=1 technical repeat).

(**F**) Representative trace of ECAR of three independent experiments in naive B cells isolated from naïve WT mice in response to stimulation in the presence or absence of IL-4+CD40 plus or minus corticosterone (n=1 technical repeat).

(**G**) NR3C1 expression in B cells of $NR3C1^{WT}$ and $NR3C1^{\Delta B\ cells}$ mice·

(**H**) Plasma anti-tumor IgG in $NR3C1^{\Delta B\ cells}$ mice(n=3 mice/group).

(**I**) Experimental layout for Abx treatment in NP-KLH immunization experiment. WT mice were given broad-spectrum antibiotics and initiated with CUMS 7 days prior to NP-KLH immunization, with a booster NP-KLH dose 7 days after the first dose and sacrifice of mice at 21 post NP-KLH immunization. Graphs show mean ± SEM. For A-D and H, each point represents one mouse. A, B, C, D: two-tailed unpaired Student's t test; H: one-way ANOVA with Tukey's multiple comparisons and E, F: two-way ANOVA with Tukey's multiple comparisons: ns=non-significant, **p*<0.05, ***p*<0.01, ****p*<0.005 and *****p*<0.0001.

Figure S3

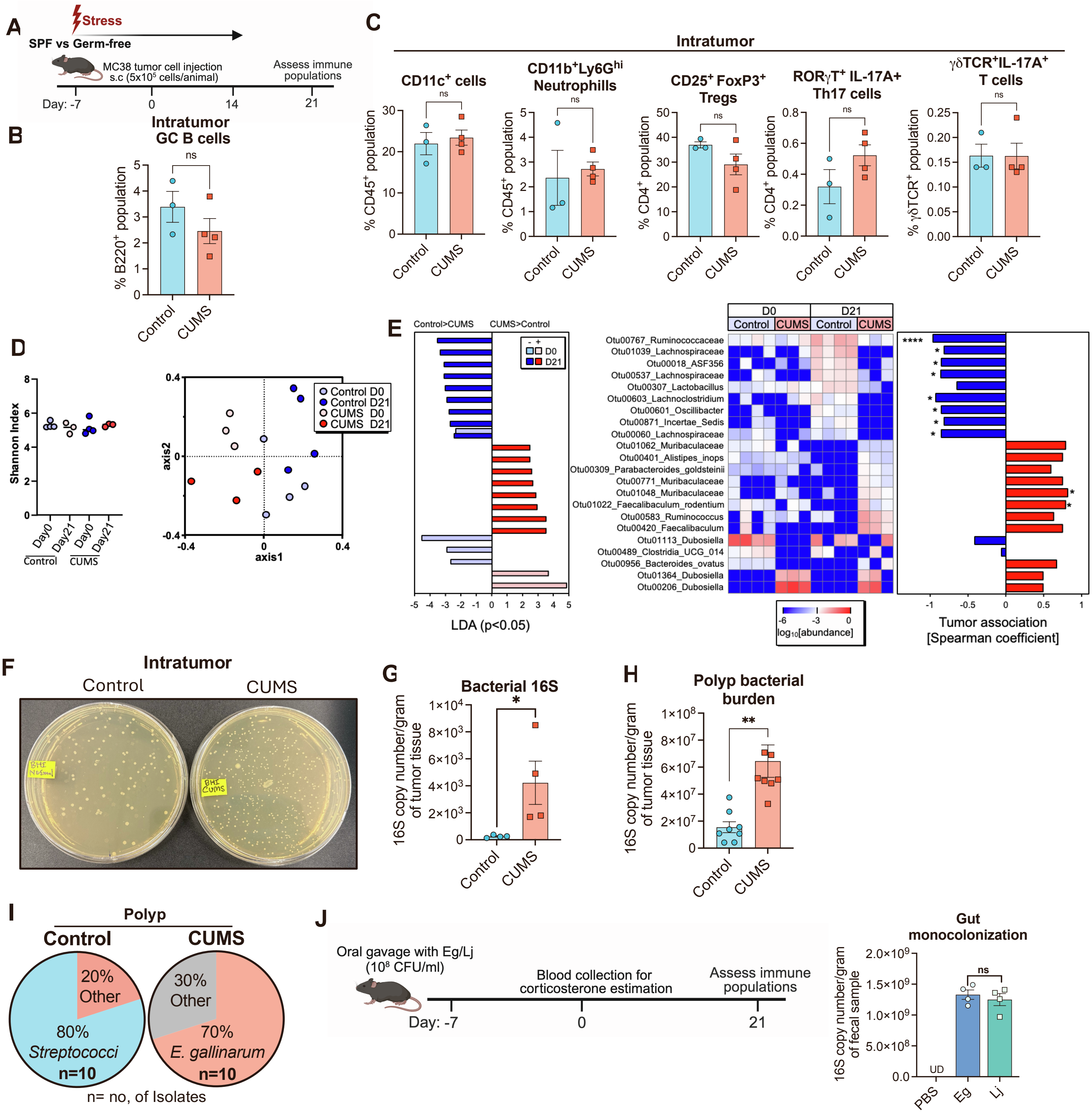
A
Stress
SPF vs Germ-free
MC38 tumor cell injection
s.c (5x10^5 cells/animal)
Assess immune populations
Day: -7
0
14
21
B
Intratumor GC B cells
% B220+ population
Control
CUMS
C
Intratumor
CD11c+ cells
CD11b+Ly6Ghi Neutrophills
CD25+ FoxP3+ Tregs
RORγT+ IL-17A+ Th17 cells
γδTCR+IL-17A+ T cells
% CD45+ population
% CD4+ population
% γδTCR+ population
D
Shannon Index
Day0
Day21
Control
CUMS
axis1
axis2
Control D0
Control D21
CUMS D0
CUMS D21
E
Control>CUMS
CUMS>Control
D0
D21
LDA (p<0.05)
Otu00767_Ruminococcaceae
Otu01039_Lachnospiraceae
Otu00018_ASF356
Otu00537_Lachnospiraceae
Otu00307_Lactobacillus
Otu00603_Lachnoclostridium
Otu00601_Oscillibacter
Otu00871_Incertae_Sedis
Otu00060_Lachnospiraceae
Otu01062_Muribaculaceae
Otu00401_Alistipes_inops
Otu00309_Parabacteroides_goldsteinii
Otu00771_Muribaculaceae
Otu01048_Muribaculaceae
Otu01022_Faecalibaculum_rodentium
Otu00583_Ruminococcus
Otu00420_Faecalibaculum
Otu01113_Dubosiella
Otu00489_Clostridia_UCG_014
Otu00956_Bacteroides_ovatus
Otu01364_Dubosiella
Otu00206_Dubosiella
log10[abundance]
Tumor association [Spearman coefficient]
F
Intratumor
Control
CUMS
G
Bacterial 16S
16S copy number/gram of tumor tissue
H
Polyp bacterial burden
I
Polyp
Control
CUMS
20% Other
80% Streptococci
n=10
30% Other
70% E. gallinarum
n=10
n= no, of Isolates
J
Oral gavage with Eg/Lj (10^8 CFU/ml)
Blood collection for corticosterone estimation
Assess immune populations
Day: -7
0
21
Gut monocolonization
16S copy number/gram of fecal sample
UD
PBS
Eg
Lj

Figure S3. CUMS stress impairs B cells and exacerbates tumor growth by modulating intratumor microbiota (related to Figure 3)

(**A**) Experimental layout of MC38 tumor model in Control and CUMS SPF and GF mice (n=3-4 mice/group).

(**B**) Intratumor percentage population of $B220^{+}GL7^{+}FAS^{+}$ GC B cells in GF control and CUMS tumors (n=3-4 mice/group).

(**C**) Intratumor innate and T cell populations in MC38 tumor-bearing GF mice (n=3-4 mice/group).

(**D**) Bar and scatter plots showing alpha and beta diversity of gut microbiota at day 0 and 21 of CUMS (n=3-4 mice/group).

(**E**) LEfSe analysis of 16S rRNA sequencing of fecal bacteria at day 0 and 21 of CUMS (n=3-4 mice/group).

(**F**) Colony density of MC38 tumor homogenates cultured on BHI agar plates.

(**G-H**) Intratumor bacterial 16S abundance in B16F10 (n=4 mice/group) and *cdx2*-Cre^{ER}*Apc*$^{fl/fl}$ mice Polyps (n=6-8 mice/group).

(**I**) Pie chart for Eg abundance in Control and CUMS polyps determined by sanger sequencing of cultured colonies (n=10 isolates/group).

(**J**) Experimental layout to demonstrate the impact of Eg at steady state. GF mice were mono-colonized with 10^{8} CFU of Eg, or *Lactobacillus johnsonii* (Lj) as a control, and were sacrificed after 21 days. Gut colonization of Eg and Lj measured by 16S qPCR copy number of fecal DNA at day 14 post colonization (n=4 mice/group).

Graphs show mean ± SEM with each point representing one mouse or technical repeat. B, C, G, H: two-tailed unpaired Student’s t test; D(left): one-way ANOVA with non-parametric Shapiro-Wilk test; D(right): PERMANOVA; E: Spearman's rank correlation coefficient; I, J: one-way ANOVA with Tukey’s multiple comparisons: ns=non-significant, **p*<0.05, ***p*<0.01, ****p*<0.005 and *****p*<0.0001.

Figure S4

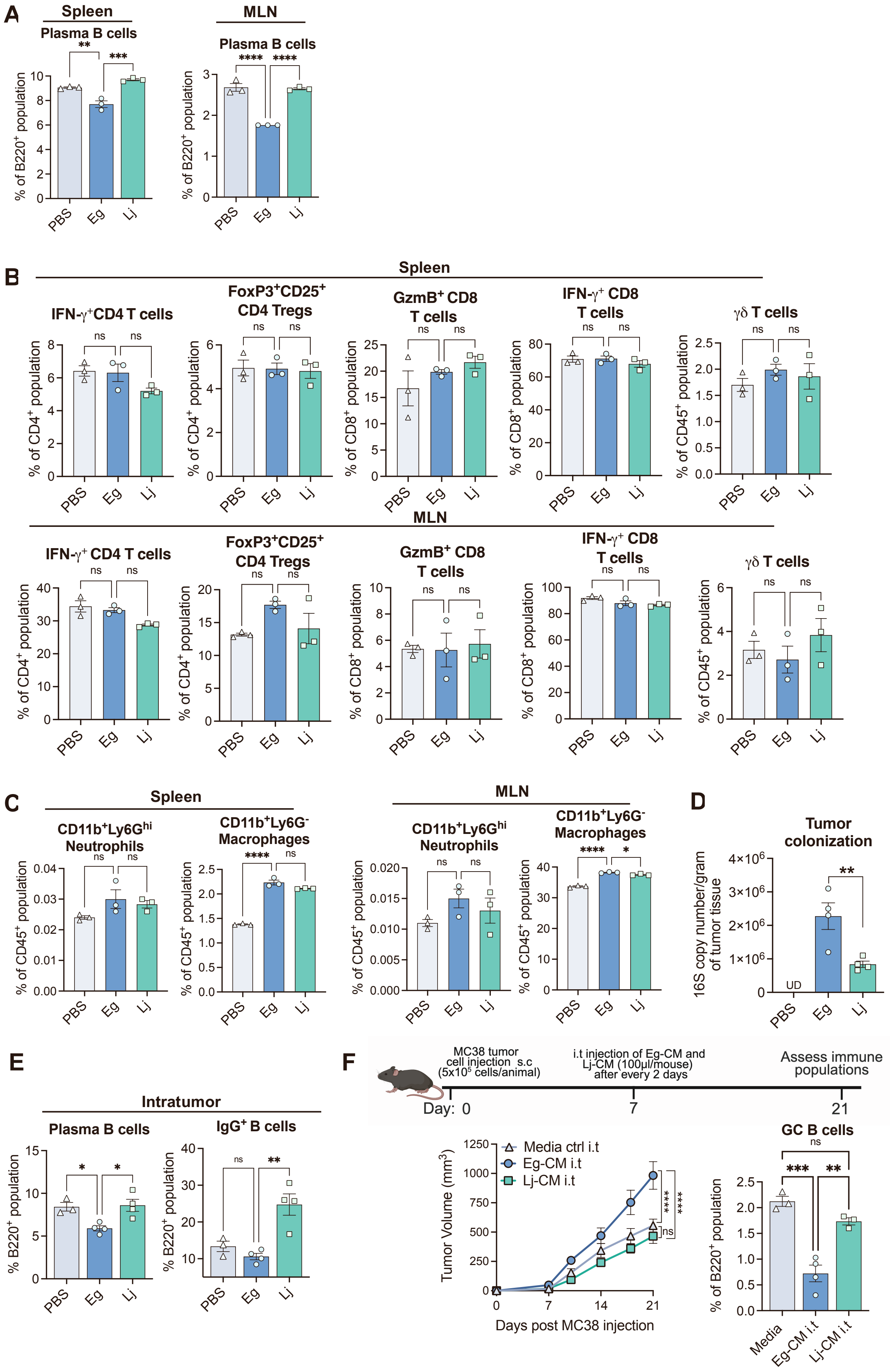

A
Spleen
Plasma B cells
% of B220+ population
MLN
Plasma B cells
PBS
Eg
Lj
B
Spleen
IFN-γ+CD4 T cells
FoxP3+CD25+ CD4 Tregs
GzmB+ CD8 T cells
IFN-γ+ CD8 T cells
γδ T cells
% of CD4+ population
% of CD8+ population
% of CD45+ population
MLN
IFN-γ+ CD4 T cells
FoxP3+CD25+ CD4 Tregs
GzmB+ CD8 T cells
IFN-γ+ CD8 T cells
γδ T cells
C
Spleen
CD11b+Ly6Ghi Neutrophils
CD11b+Ly6G- Macrophages
MLN
CD11b+Ly6Ghi Neutrophils
CD11b+Ly6G- Macrophages
% of CD45+ population
D
Tumor colonization
16S copy number/gram of tumor tissue
UD
E
Intratumor
Plasma B cells
IgG+ B cells
% B220+ population
F
MC38 tumor cell injection s.c (5x10^5 cells/animal)
i.t injection of Eg-CM and Lj-CM (100µl/mouse) after every 2 days
Assess immune populations
Day: 0
7
21
Media ctrl i.t
Eg-CM i.t
Lj-CM i.t
Tumor Volume (mm3)
Days post MC38 injection
GC B cells
% of B220+ population
Media
Eg-CM i.t
Lj-CM i.t

Figure S4. Eg enhances tumor progression via induction of corticosterone to specifically dampen anti-tumor B cell response (related to Figures 3 and 4)

(**A**) $B220^+CD38^+IgD^-$ plasma B cell population in spleen and MLN in mice gavaged with Eg and Lj (n=3 mice/group).

(**B-C**) Different T and innate cell populations in spleen and MLN in Eg- and Lj-monocolonized mice (n=3 mice/group).

(**D**) Intratumor abundance of Eg and Lj measured by 16S qPCR (n=4 mice/group).

(**E**) Intratumor $B220^+CD38^+IgD^-$ plasma and $B220^+IgG^+$ B cells (n=3-4 mice/group).

(**F**) MC38 tumor growth kinetics and intratumor GC B cells in GF mice intratumorally injected twice a week with BHI media (Control), Eg-conditioned, or Lj-conditioned media (n=3 mice/group).

Graphs show mean ± SEM with each point representing one mouse. A, B,C, D, E, F(right): one-way ANOVA with Tukey's multiple comparisons and F(left): two-way ANOVA with Tukey's multiple comparisons: ns=non-significant, *p<0.05, **p<0.01, ***p<0.005 and ****p<0.0001.

Figure S5

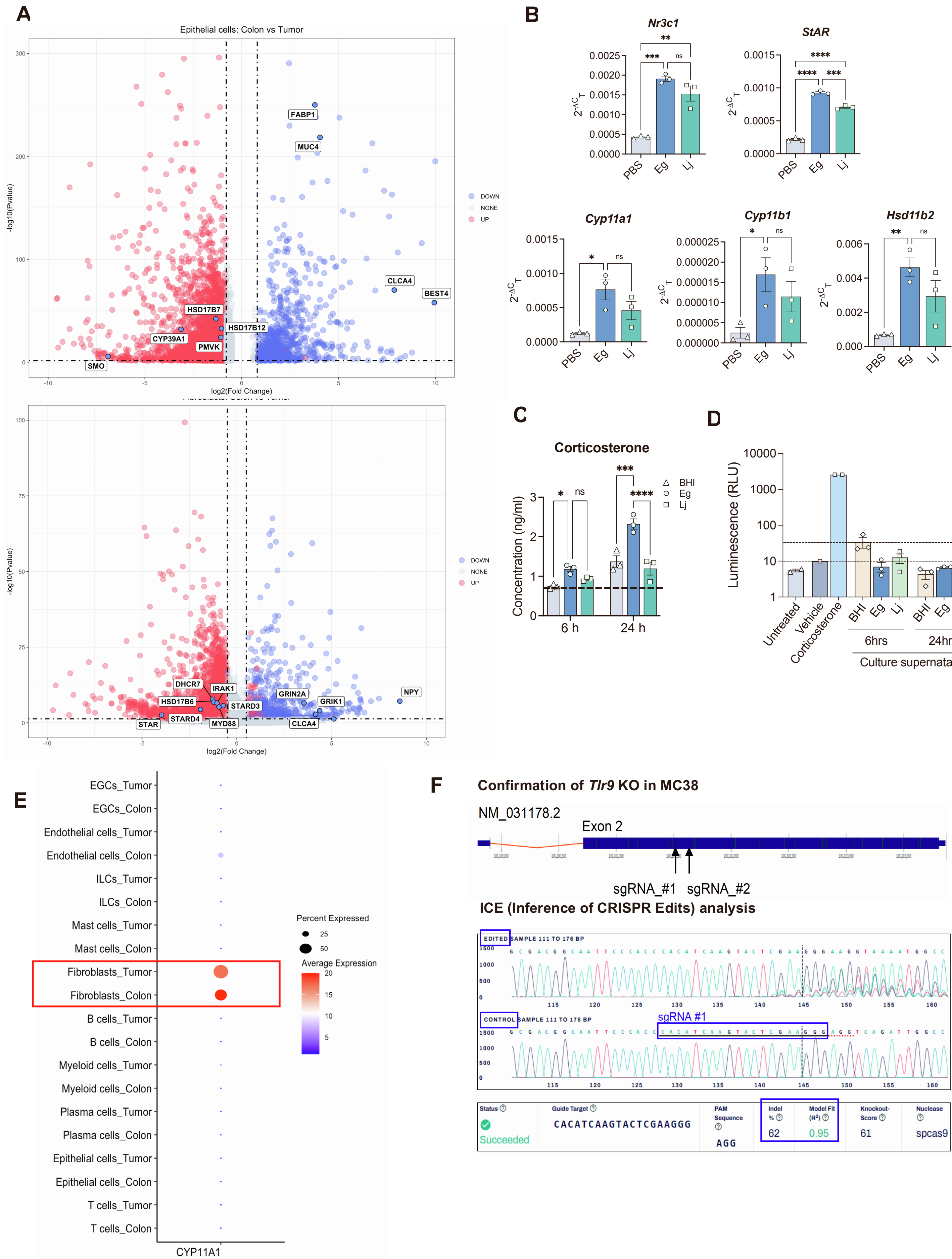

A
Epithelial cells: Colon vs Tumor
-log10(Pvalue)
log2(Fold Change)
DOWN
NONE
UP
FABP1
MUC4
CLCA4
BEST4
HSD17B7
HSD17B12
CYP39A1
PMVK
SMO
Fibroblasts: Colon vs Tumor
DHCR7
IRAK1
HSD17B6
STARD3
STARD4
MYD88
STAR
GRIN2A
GRIK1
CLCA4
NPY
B
Nr3c1
StAR
Cyp11a1
Cyp11b1
Hsd11b2
$2^{-\Delta C_T}$
PBS
Eg
Lj
ns
C
Corticosterone
Concentration (ng/ml)
BHI
Eg
Lj
6 h
24 h
D
Luminescence (RLU)
Untreated
Vehicle
Corticosterone
BHI
Eg
Lj
6hrs
24hrs
Culture supernatant
E
EGCs_Tumor
EGCs_Colon
Endothelial cells_Tumor
Endothelial cells_Colon
ILCs_Tumor
ILCs_Colon
Mast cells_Tumor
Mast cells_Colon
Fibroblasts_Tumor
Fibroblasts_Colon
B cells_Tumor
B cells_Colon
Myeloid cells_Tumor
Myeloid cells_Colon
Plasma cells_Tumor
Plasma cells_Colon
Epithelial cells_Tumor
Epithelial cells_Colon
T cells_Tumor
T cells_Colon
CYP11A1
Percent Expressed
25
50
Average Expression
F
Confirmation of Tlr9 KO in MC38
NM_031178.2
Exon 2
sgRNA_#1
sgRNA_#2
ICE (Inference of CRISPR Edits) analysis
EDITED SAMPLE 111 TO 176 BP
CONTROL SAMPLE 111 TO 176 BP
sgRNA #1
Status
Succeeded
Guide Target
CACATCAAGTACTCGAAGGG
PAM Sequence
AGG
Indel %
62
Model Fit (R^2)
0.95
Knockout-Score
61
Nuclease
spcas9

Figure S5. Eg directly induces the extra-adrenal corticosterone pathway (related to Figure 4)
(**A**) Differential gene expression in epithelial cells and fibroblasts from human colon tumors and adjacent normal colon tissue; data taken from a publicly-available scRNA-seq dataset (n=17 patients/group).
(**B**) Differential expression of extra-adrenal genes in intestinal organoids derived from Eg- and Lj-monocolonized GF mice, where gene expression was quantified by qRT-PCR using the comparative Ct method. Ct values of target genes were first normalized to the housekeeping gene GAPDH for each sample by calculating ΔCt values (n=3 mice/group).
(**C**) Corticosterone level in bacterial culture supernatants at different time points (n=3 technical repeats/group).
(**D**) Relative luminescence of NR3C1 reporter cell line stimulated with different Eg and Lj culture supernatants with corticosterone as positive controls (n=3 technical repeats/group).
(**E**) Differential expression of extra-adrenal genes in fibroblasts from human colon tumors and adjacent normal colon tissue, based on reanalysis of a publicly available scRNA-seq dataset with color and size of circle depicting average expression and percent expressed, respectively (n=17 patients/group).
(**F**) Confirmation of TLR9 KO MC38 cells using sequence deconvolution tools (ICE and TIDE) showing 62% efficiency of biallelic disruption of Tlr9 in MC38 cells.
Graphs show mean ± SEM. For B, each point representes one mouse and C-D each point represents one technical replicate. B: one-way ANOVA with Tukey's multiple comparisons and C, D: two-way ANOVA with Tukey's multiple comparisons: ns=non-significant, *p<0.05, **p<0.01, ***p<0.005 and ****p<0.0001.

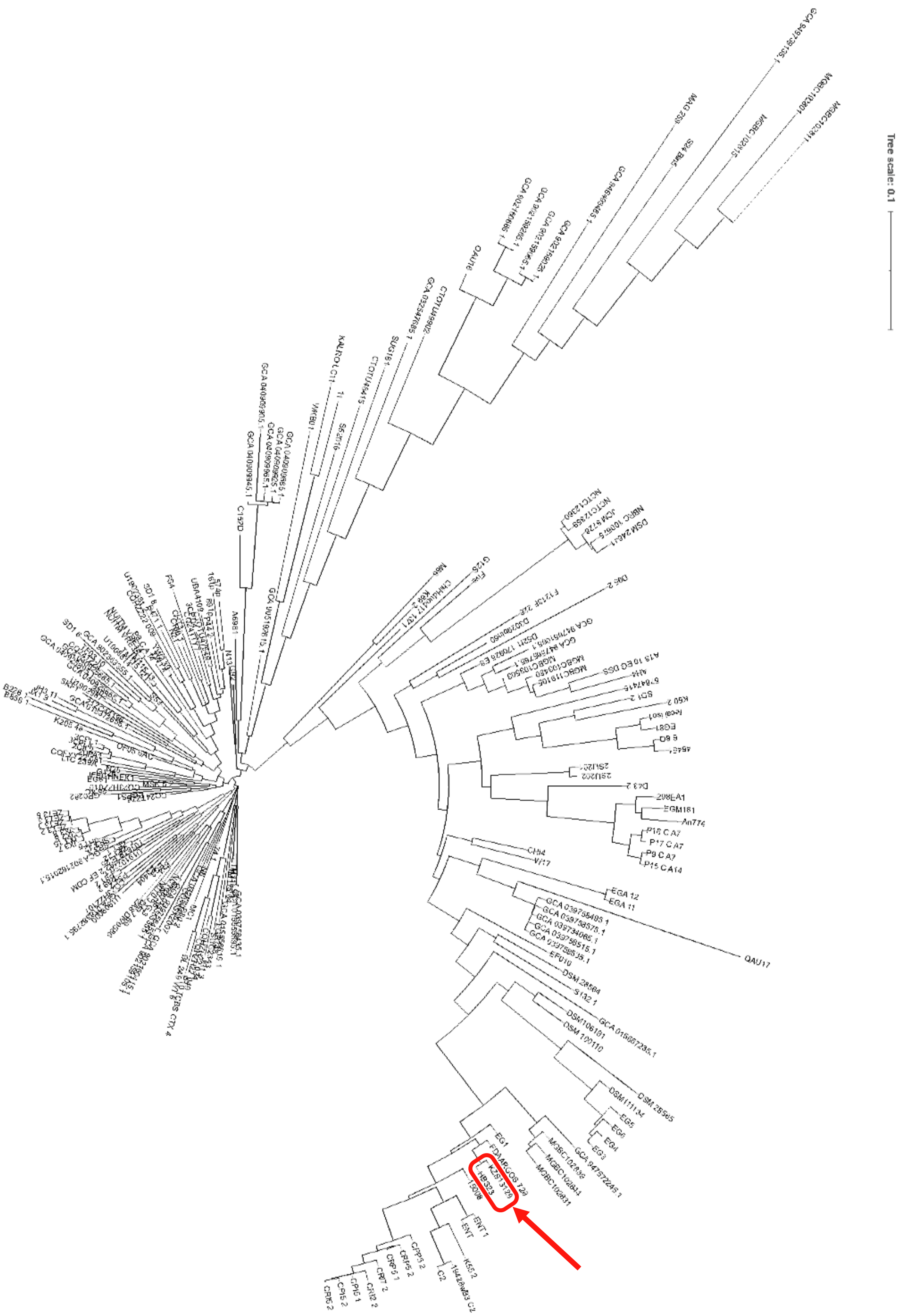

Figure S6. Eg-Tum evolutionary divergence from other Eg strains. (related to Figure 5)
Phylogenetic analysis of highlighted Eg-Tum (HBS23) showing evolutionary divergence from other Eg strains including Eg-Gut (KZS13129). Phylogenetic tree was generated by iTol and using roary (default parameters).

Figure S7

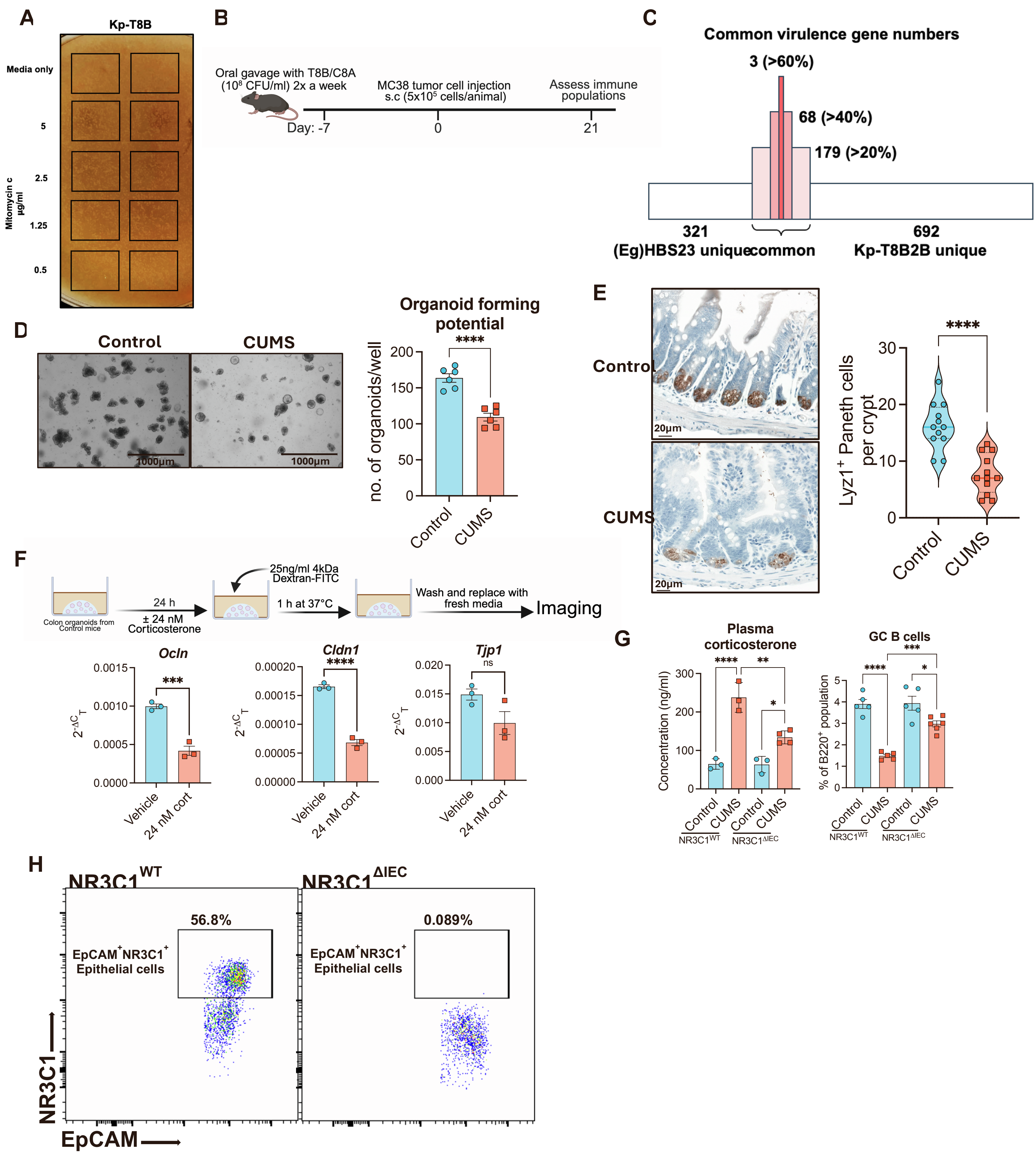

A
Kp-T8B
Media only
5
2.5
1.25
0.5
Mitomycin c µg/ml
B
Oral gavage with T8B/C8A (10^8 CFU/ml) 2x a week
MC38 tumor cell injection s.c (5x10^5 cells/animal)
Assess immune populations
Day: -7
0
21
C
Common virulence gene numbers
3 (>60%)
68 (>40%)
179 (>20%)
321 (Eg)HBS23 unique
common
692 Kp-T8B2B unique
D
Control
CUMS
1000µm
1000µm
Organoid forming potential
no. of organoids/well
****
Control
CUMS
E
Control
CUMS
20µm
20µm
Lyz1+ Paneth cells per crypt
****
Control
CUMS
F
Colon organoids from Control mice
24 h
± 24 nM Corticosterone
25ng/ml 4kDa Dextran-FITC
1 h at 37°C
Wash and replace with fresh media
Imaging
Ocln
***
Cldn1
****
Tjp1
ns
2-ΔCT
Vehicle
24 nM cort
G
Plasma corticosterone
Concentration (ng/ml)
**** ** *
GC B cells
% of B220+ population
**** *** *
Control
CUMS
NR3C1WT
NR3C1ΔIEC
H
NR3C1WT
NR3C1ΔIEC
56.8%
0.089%
EpCAM+NR3C1+ Epithelial cells
NR3C1
EpCAM

Figure S7. IEC-intrinsic glucocorticoid signaling linking gut barrier defects to anti-tumor B cell responses (related to Figures 5 and 6)

(**A**) Lawn colony plaque formation of Kp-T8B following treatment with mitomycin C to induce the release of phages.

(**B**) Experimental layout: mice orally gavaged twice a week with PBS (control), Kp-T8B, or Kp-C8A (100ul of 10^8 CFU/ml).

(**C**) The homology of Eg-Tum and Kp-T8B compared with virulence factors database (VFDB) and determined by BLASTP showing common virulence genes like *Clp* protease, *lap* and *sigA* .

(**D**) Intestinal organoids were generated from small intestinal crypts of Control and day-7 CUMS mice and after 4-5 days of ex vivo culture in differentiation media, organoids were counted in each well to determine organoid forming potential of intestinal crypts derived from Control and CUMS mice (n=6 mice/group).

(**E**) Histochemical images of Lyz1$^+$ Paneth cells at the base of the intestinal crypts in day-7 Control and CUMS mice and quantification of Lyz1$^+$ Paneth cells per crypt (n=3 mice/group).

(**F**) Experimental strategy for in vitro colon organoid Dextran-FITC permeability assay and qPCR based expression of tight junction genes *Ocln*, *Cldn1* and *Tjp1* (n=3 technical repeats/group).

(**G**) Plasma corticosterone and intratumor GC B cells in NR3C1^{WT} and intestinal epithelial cell specific NR3C1 deficient (NR3C1$^{\Delta IECs}$) MC38 tumor bearing mice (n=3-5 mice/group).

(**H**) NR3C1 expression in intestinal epithelial (EpCAM$^+$CD31$^-$PDPN$^-$CD45$^-$) cells of NR3C1^{WT} and NR3C1$^{\Delta IEC}$ mice.

Graphs show mean ± SEM. For D and G each point represents one mouse and for F each point represents one technical replicate. D, F: two-tailed unpaired Student's t test; E: unpaired t test with Welch's correction and G: one-way ANOVA with Tukey's multiple comparisons: ns=non-significant, *p<0.05, **p<0.01, ***p<0.005 and ****p<0.0001.

Figure S8

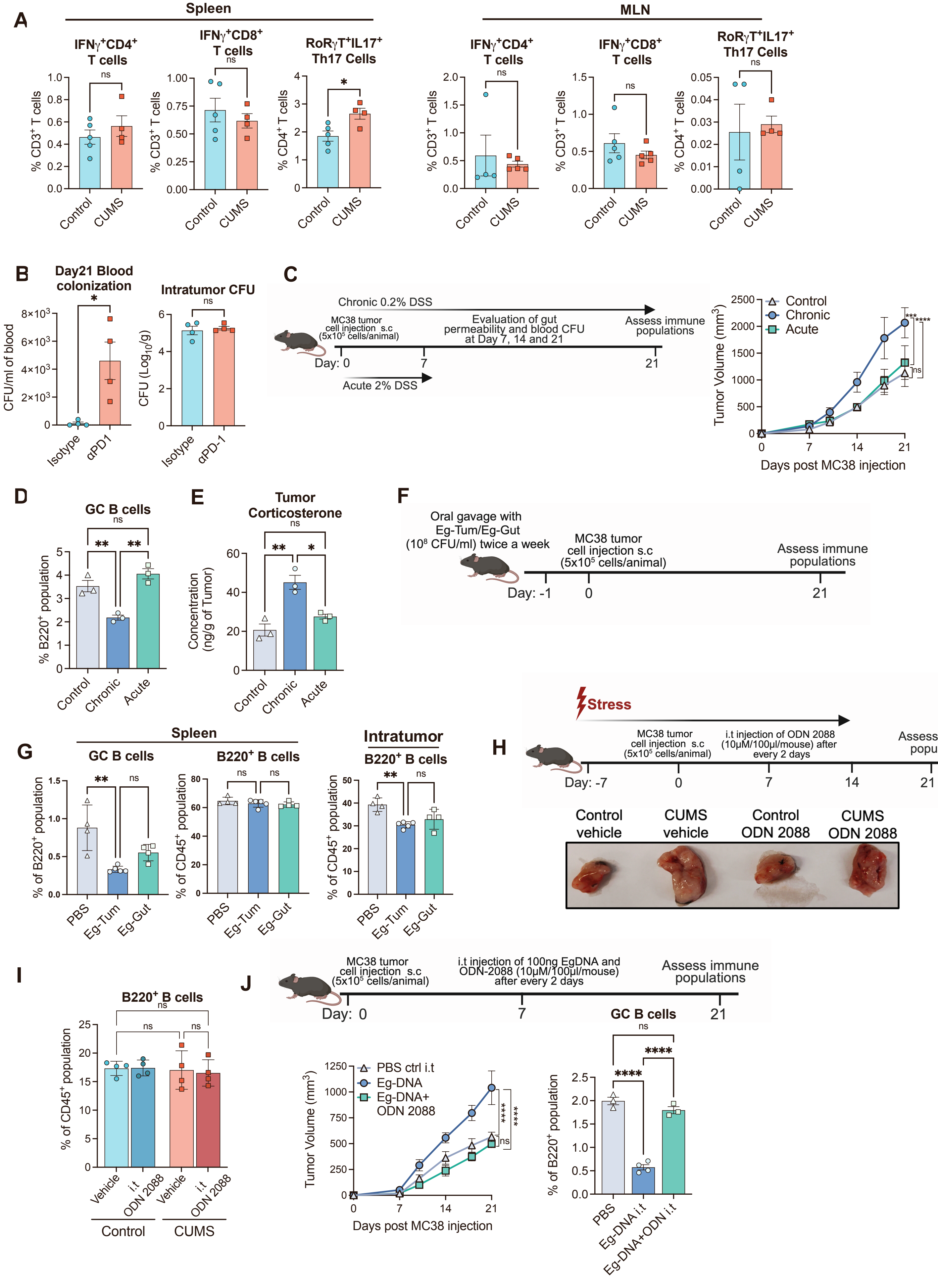

A
Spleen
MLN
IFNγ+CD4+ T cells
IFNγ+CD8+ T cells
RoRγT+IL17+ Th17 Cells
% CD3+ T cells
% CD4+ T cells
Control
CUMS
ns
*
B
Day21 Blood colonization
Intratumor CFU
CFU/ml of blood
CFU (Log10/g)
Isotype
αPD1
αPD-1
C
Chronic 0.2% DSS
MC38 tumor cell injection s.c (5x10^5 cells/animal)
Evaluation of gut permeability and blood CFU at Day 7, 14 and 21
Assess immune populations
Day: 0
7
21
Acute 2% DSS
Control
Chronic
Acute
Tumor Volume (mm3)
Days post MC38 injection
D
GC B cells
% B220+ population
E
Tumor Corticosterone
Concentration (ng/g of Tumor)
F
Oral gavage with Eg-Tum/Eg-Gut (10^8 CFU/ml) twice a week
MC38 tumor cell injection s.c (5x10^5 cells/animal)
Assess immune populations
Day: -1
0
21
G
Spleen
GC B cells
B220+ B cells
Intratumor
B220+ B cells
% of B220+ population
% of CD45+ population
PBS
Eg-Tum
Eg-Gut
H
Stress
MC38 tumor cell injection s.c (5x10^5 cells/animal)
i.t injection of ODN 2088 (10μM/100μl/mouse) after every 2 days
Day: -7
0
7
14
21
Control vehicle
CUMS vehicle
Control ODN 2088
CUMS ODN 2088
I
B220+ B cells
% of CD45+ population
Vehicle
i.t ODN 2088
Control
CUMS
J
MC38 tumor cell injection s.c (5x10^5 cells/animal)
i.t injection of 100ng EgDNA and ODN-2088 (10μM/100μl/mouse) after every 2 days
Assess immune populations
Day: 0
7
21
PBS ctrl i.t
Eg-DNA
Eg-DNA+ ODN 2088
Tumor Volume (mm3)
Days post MC38 injection
GC B cells
% of B220+ population
PBS
Eg-DNA i.t
Eg-DNA+ODN i.t

Figure S8. Targeting intratumor TLR9 or bacteria reverses the effect of CUMS on tumor growth (related to Figures 6 and 7)

(**A**) Different T cell populations in spleen and MLN comparing Control and CUMS mice (n=4-5 mice/group).

(**B**) Day 21 blood bacterial CFU and Intratumor CFU in MC38 tumor bearing WT mice receiving α-PD1 and isotype control antibody (n=4 mice/group).

(**C**) Experimental layout for chronic and acute DSS treatment (ad-libitum) in mice as well as MC38 tumor growth kinetics (n=3 mice/group).

(**D-E**) Intratumor GC B cell population and intratumor corticosterone level (n=3 mice/group).

(**F**) Experimental layout: WT SPF mice were orally gavaged with 100ul of 10^8 CFU/ml of Eg-Tum or Eg-Gut twice a week until the end of experiment, with MC38 tumor cells injected s.c. on 1 day post first oral gavage.

(**G**) Splenic GC B cell and total B cell populations and intratumor B cells, 21 days post tumor inoculation in mice gavaged with Eg-Tum and Eg-gut (n=4-5 mice/group).

(**H**) Experimental strategy for intratumor injection of ODN 2088 (10μM) in Control and CUMS MC38 tumor bearing mice as well as representative images of Day 21 tumors in Control and CUMS mice in presence or absence of ODN 2088.

(**I**) Total intratumor $B220^+$ B cells in presence or absence of ODN 2088 in Control and CUMS mice (n=4 mice/group). WT mice were injected with MC38 tumor cells at day 0 and from day7, EgDNA (100ng/mice) was intratumorally injected twice a week in presence and absence and absence of ODN 2088 (10μM) throughout the experiment.

(**J**) Experimental strategy for intratumor injection of EgDNA in MC38 tumor bearing mice, tumor growth kinetics and intratumor GC B cell population (n=3-4 mice/group).

Graphs show mean ± SEM. For A-B and D-J, each point represents one mouse. A, B: two-tailed unpaired Student's t test; D, E, G, I, J(right): one-way ANOVA with Tukey's multiple comparisons; C, J(left): two-way ANOVA with Tukey's multiple comparisons: ns=non-significant, *p<0.05, **p<0.01, ***p<0.005 and ****p<0.0001.

Table S1. (related to STAR Methods)

**qPCR primers related to Figures 6, S5 and S7**	**Sequence**	
mStAR-forward	ATGTTCCTCGCTACGTTCAAG	
mStAR-reverse	CCCAGTGCTCTCCAGTTGAG	
mCyp11a1-forward	AGGTCCTTCAATGAGATCCCTT	
mCyp11a1- reverse	TCCCTGTAAATGGGGCCATAC	
mCyp11b1-forward	TTGTGTTTGTGACGTTGCCG	
mCyp11b1- reverse	AGCTGCAGTCGGTTGAAGTA	
mHsd11b2-forward	GGTTGTGACACTGGTTTTGGC	
mNr3c1-forward	GGCCTTATCTGCTAGAAGTGGG	
mNr3c1- reverse	TTGACTGTAGCTCCACCCCT	
mGapdh-forward	CAACTCCCTCAAGATTGTCAGCAA	
mGapdh- reverse	GGCATGGACTGTGGTCATGA	
mLgr5-forward	CAGGTCAATACCGGAGCGAG	
mLgr5- reverse	GCGAGGCACCATTCAAAGTC	
mLyz1-forward	TCTACAATCGTTGTGAGTTGGC	
mLyz1-reverse	ATAGTCGGTGCTTCGGTCTC	
mOcln-forward	TTGAAAGTCCACCTCCTTACAGA	
mOcln-reverse	CCGGATAAAAAGAGTACGCTGG	
mCldn1- forward	GGGGACAACATCGTGACCG	
mCldn1- reverse	AGGAGTCGAAGACTTTGCACT	
mTjp1- forward	GCCGCTAAGAGCACAGCAA	
mTjp1- reverse	TCCCCACTCTGAAAATGAGGA	
**Bacterial primers related to Figures 3, S3 and S4**	**Sequence**	
16S - 8F	AGAGTTTGATCCTGGCTCAG	
16S - 1492R	CGGTTACCTTGTTACGACTT	
16S (V3-V4)-forward	CCTACGGGTGGCTGCAG	
16S (V3-V4)-reverse	GACTACTAGGGTATCTAATCC	
**Oligonucleotides related to Figures 4 and S5**		
sgNon-Targeting Oligo1	Bionics Korea	CACCGGCGAGGTATTCGGCTCCGCG
sgNon-Targeting Oligo2	Bionics Korea	AAACCGCGGAGCCGAATACCTCGCC
Tlr9-KO sgRNA #1 Oligo1	Bionics Korea	CACCGCACATCAAGTACTCGAAGGG
Tlr9-KO sgRNA #1 Oligo2	Bionics Korea	AAACCCCTTCGAGTACTTGATGTGC
Tlr9-KO sgRNA #2 Oligo1	Bionics Korea	CACCGGTCCTTCAATTACCGCAAGA
Tlr9-KO sgRNA #2 Oligo2	Bionics Korea	AAACTCTTGCGGTAATTGAAGGACC
